# Intracellular Molecular Targets and Signaling Pathways Involved in Antioxidative and Neuroprotective Effects of Cannabinoids in Neurodegenerative Conditions

**DOI:** 10.3390/antiox11102049

**Published:** 2022-10-18

**Authors:** Ana Tadijan, Ignacija Vlašić, Josipa Vlainić, Domagoj Đikić, Nada Oršolić, Maja Jazvinšćak Jembrek

**Affiliations:** 1 Division of Molecular Medicine, Ruđer Bošković Institute, Bijenička 54, HR-10000 Zagreb, Croatia; 2 Division of Animal Physiology, Faculty of Science, University of Zagreb, Rooseveltov Trg 6, HR-10000 Zagreb, Croatia; 3 School of Medicine, Catholic University of Croatia, Ilica 242, HR-10000 Zagreb, Croatia

**Keywords:** cannabinoids, neuroprotection, redox-sensitive signaling pathways, oxidative stress, neuroinflammation

## Abstract

In the last few decades, endocannabinoids, plant-derived cannabinoids and synthetic cannabinoids have received growing interest as treatment options in neurodegenerative conditions. In various experimental settings, they have displayed antioxidative, anti-inflammatory, antiapoptotic, immunomodulatory, and neuroprotective effects. However, due to numerous targets and downstream effectors of their action, the cellular and molecular mechanisms underlying these effects are rather complex and still under discussion. Cannabinoids are able to neutralize free radicals and modulate the production of reactive oxygen species and the activity of antioxidative systems acting on CB1 and CB2 cannabinoid receptors. The activation of CB1 receptors stimulates signaling pathways involved in antioxidative defense and survival (such as the phosphoinositide 3-kinase (PI3K)/Akt, mitogen-activated protein kinase (MAPK), and Nrf2 pathways) and regulates glutamatergic signaling, the activation of N-methyl-D-aspartate (NMDA) receptors, calcium influx, and the induction of Ca^2+^-regulated signaling cascades, whereas the neuroprotective effects mediated by CB2 receptors are due to the suppression of microglial activation and the release of prooxidative and proinflammatory mediators. This review summarizes the main molecular mechanisms and new advances in understanding the antioxidative and neuroprotective effects of cannabinoids. Because of the plethora of possible pharmacological interventions related to oxidative stress and cannabinoid-mediated neuroprotection, future research should be directed towards a better understanding of the interplay between activated signal transduction pathways and molecular targets with the aim to improve treatment options and efficacy by targeting the endocannabinoid system.

## 1. Introduction

*Cannabis* is one of the first plants that was cultivated for human use. The earliest writings about the medical uses of *Cannabis* can be found in Chinese pharmacopoeias as early as second century BCE [1]. In 1980, epidemiological studies described the potential anticonvulsant effects of marijuana extracts. Since then, a great amount of research has been conducted to better reveal the pharmacological effects of natural and synthetic cannabinoids. These studies demonstrated that they could exert many beneficial health effects. The neuroprotective potential of cannabinoids has been investigated in a wide range of brain-related diagnoses. These include brain tumors, neurodegeneration-related diseases, multiple sclerosis, neuropathic pain, and some specific forms of childhood seizures [2,3,4]. The therapeutic potential of cannabinoids is also being considered for various psychiatric diseases such as schizophrenia, anxiety, autism, addiction, and depression [5,6].

### The Endocannabinoid System

The lipid endocannabinoid system (ECS) consists of G protein-coupled cannabinoid receptors (GPCRs) CB1 and CB2, endogenous cannabinoids (endocannabinoids), and enzymes involved in their synthesis and metabolism [7,8,9]. Both types of cannabinoid receptors inhibit adenylyl cyclase and protein kinase A (PKA) and modulate the activation of mitogen-activated protein kinases (MAPKs) (including extracellular signal-regulated kinases (ERKs), c-Jun N-terminal kinases (JNKs), and p38 kinases) via G_i/o_ signaling [10]. It is considered that CB1 and CB2 receptors regulate various aspects of neuronal physiology, acting independently and/or cooperatively [7,11].

Besides CB1 and CB2 receptors, additional receptors are involved in the biological effects of cannabinoids with signaling distinct from the CB1 and CB2 receptors, including the nuclear peroxisome proliferator-activated receptors (PPARs), transient receptor potential vanilloid type 1 (TRPV1) channel, G protein-coupled receptor 55 (GPR55) and G protein-coupled receptor 119 (GPR119), metabotropic glutamate receptors, µ- and δ-opioid receptors, and serotonin 1A receptor (5HT1A) [10,12,13,14,15,16,17,18,19,20].

The best-characterized endocannabinoids that act via the CB1 and CB2 receptors are eicosanoids *N*-arachidonoyl ethanolamide (anandamide) and 2-arachidonoyl glycerol (2-AG). These small lipid transmitters are synthesized on demand from the membrane phospholipids that contain arachidonic acid. They are produced by specific lipases as a response to increased intracellular Ca^2+^ levels, and it is generally considered that they are immediately released, without storage in the vesicles [9,10,21]. Both display a higher relative intrinsic affinity for CB1 receptors than for CB2 receptors. 2-AG is a full agonist, whereas anandamide behaves as a partial agonist of the two cannabinoid receptors [8,10,21,22].

The inactivation of cannabinoids includes cellular uptake and hydrolysis. Anandamide is degraded by fatty acid amide hydrolase (FAAH) to arachidonic acid and glycerol. Some other enzymes, such as cyclooxygenase-2 (COX2) and lipoxygenases, that are upregulated during neuroinflammation are also able to metabolize anandamide, some of them providing derivatives that may promote endocannabinoid-like effects [7,8]. The COX-2 metabolism of anandamide generates anandamide-derived prostaglandins (prostaglandin-ethanolamides or PGs-EA) that are relatively poor activators of CB1 and CB2 receptors. 12- or 15-lipoxygenases convert anandamide into 12(S)-hydroxyeicosatetraenoic acid-ethanolamine (HETE)-EA and 15(S)-HETE-EA that target CB1 receptors [23,24,25]. Several cytochrome P450 isoforms also metabolize anandamide to hydroxylated and epoxygenated metabolites. The oxidation of anandamide by human cytochrome P450 enzymes yields metabolites such as 20-hydroxyeicosatetraenoic acid ethanolamide and the 5,6-, 8,9-, 11,12-, and 14,15-epoxyeicosatrienoic acid ethanolamides. Pharmacological studies have shown that 20-hydroxyeicosatetraenoic acid ethanolamide (20-HETE-EA) and 14,15-epoxyeicosatetraenoic acid ethanolamide (14,15-EET-EA) bind to rat CB1 receptors [26], whereas the 5,6-epoxide of anandamide, 5,6-epoxyeicosatrienoic acid ethanolamide (5,6-EET-EA), is a potent and selective agonist of CB2 receptors [27]. Diacylglycerol (DAG), the product of phospholipase C (PLC), is the main precursor for 2-AG synthesis. DAG lipase (DAGL) catalyzes the hydrolysis of DAG and forms 2-AG, which is further converted into arachidonic acid and glycerol, mostly by the activity of the serine hydrolase monoacylglycerol lipase (MAGL). 2-AG may also be a substrate for COX and lipoxygenases.

In the central nervous system (CNS), endocannabinoids can be produced and degraded by both neurons and glia [9,28,29,30,31]. It has been shown that reactive microglia secrete hydrophobic anandamide in the form of extracellular vesicles, i.e., microvesicles, through the outward blebbing of the microglial plasma membrane or as exosomes formed in the endosomal system. In microvesicles, anandamide is carried on their surface and is able to stimulate CB1 receptors in target neurons [32,33]. On the other hand, adiposomes, the lipid droplets, represent an intracellular reservoir for the accumulation of taken up anandamide. These lipid droplets are spatially associated with anandamide hydrolase, and adiposome size correlates with the intensity of anandamide catabolism. Although these findings may challenge the dogma that anandamide is produced on demand, the biological context of anandamide storage needs to be addressed in further studies [34,35].

CB1 receptors are the main targets of endocannabinoids and are the most abundant GPCRs in the brain. They are primarily located at presynaptic terminals. The net result of endogenous cannabinoid signaling after the activation of presynaptic CB1 receptors is the inhibition of excitatory and inhibitory neurotransmission through the inhibition of neurotransmitter release (GABA, glutamate, dopamine, norepinephrine, serotonin, and acetylcholine) [7,36,37]. CB1 receptors located at post-synaptic sites regulate the activity of specific ion channels, of which ionotropic glutamate NMDA receptors have received particular attention in the context of cannabinoid-mediated and antioxidative-based neuroprotection [8,38].

CB1 receptors are abundantly expressed in most brain areas, including the prefrontal cortex, cingulate gyrus, CA3 region and dentate gyrus in the hippocampus, basal ganglia, hypothalamus, amygdala, and cerebellum, implying the important role of the ECS in cognition, motoric functions, and emotions [4,8,9,39,40]. CB1 receptors and endocannabinoids are also involved in the regulation of adult hippocampal neurogenesis and may facilitate the induction of long-term potentiation in the hippocampus [41,42,43]. Both in culture and in vivo, it was shown that anandamide may inhibit neuronal differentiation (from cortical neuron progenitors to mature neurons) via CB1 receptors, though without affecting neuronal viability [44]. The ERK-mediated phosphorylation of the transcription factor Elk is critical for the transcriptional regulation of neuronal differentiation. Anandamide attenuates the nerve growth factor (NGF)-mediated activation of the Rap1/B-Raf pathway, thereby suppressing the activation of ERK and the further phosphorylation of Elk, ultimately interfering with the differentiation program [44]. In addition to neuronal cells, the expression of CB1 receptors has been confirmed in astrocytes, oligodendrocytes, and endothelial vascular cells of the blood–brain barrier [45,46,47,48]. Outside the CNS, CB1 receptors are expressed in the peripheral and enteric nervous system [48,49,50,51]. In addition to orthosteric sites, there are one or more allosteric sites at the CB1 receptors. Ligands of these allosteric sites may modulate the activation induced by direct cannabinoid agonists and modify their effects [10].

CB2 receptors are mainly involved in immune functions and are predominantly expressed by immune cells. In physiological conditions, their expression in the brain is very low (they are detectable in brainstem neurons and the spinal cord). However, they are highly upregulated during the neuroinflammatory response that accompanies neurodegenerative diseases due to the activation and proliferation of microglial cells [52,53]. For example, in malonate-induced toxicity in rats, a marked increase in CB2 receptors in astrocytes and microglia was observed, probably as a mechanism of protection for reducing neuronal damage [54]. Similar to CB1 receptors, CB2 receptor signaling inhibits adenylyl cyclase and reduces cAMP levels and PKA activity. It has also been observed in some studies that Gα_i/o_, likely via the Gβγ subunit, may stimulate cAMP synthesis and activate the Akt and ERK pathways, presumably by regulating various adenylyl cyclase isozymes [8,53,55,56]. The activation and increased expression of CB2 receptors have been shown in various neurodegenerative diseases, and these receptors have been intensively studied as possible pharmacological targets against neuroinflammation and neuroinflammation-related neurodegeneration [54,57,58]. The observed neuroprotective effects of CB2 receptor agonists and CB2 receptor activation are mainly related to the suppression of microglial activation, the modulation of cytokine release, and the production of reactive oxygen species (ROS) [10,38,54,59,60,61]. Inflammatory mediators, such as nitric oxide (NO), ROS, proinflammatory cytokines and chemokines, are important contributing factors in microglia-mediated neuronal death due to the induction of nitrosative and oxidative stress [62]. The stimulation of CB2 receptors suppresses microglial activation via different signaling pathways, such as the Janus kinase (JAK)/signal transducer and activator of transcription 1 (STAT1) pathway [63] and the protein kinase C (PKC) pathway [64]. Moreover, 2-AG and anandamide signaling may polarize microglia towards the M2 (reparative) phenotype [29].

## 2. Intracellular Signaling Pathways Induced by Activation of CB1 and CB2 Receptors

The activation of CB1 receptors may activate different signaling cascades in three spatio-temporal waves: the first one is mediated by the activation of heterotrimeric G proteins, the second one is mediated by β-arrestins, and the third one that occurs in intracellular compartments could be initiated by both G proteins and β-arrestins [65]. The main signaling cascade activated by CB1 receptors starts with the pertussis toxin (PTX)-sensitive Gα_i/o_ protein. As already mentioned, this pathway inhibits adenylyl cyclase activity, reduces the intracellular levels of cAMP, and further modulates intracellular signaling pathways [21,66,67]. The key enzyme regulated by cAMP is PKA. Its reduced activity, due to the reduced production of cAMP, affects downstream signaling cascades and biochemical events [7,10]. However, the role of PKA in the effects of cannabinoids is not consistent. The stimulation of adenylate cyclase activity and an increase in cAMP levels have also been observed after CB1 receptor activation [8,56]. Likewise, PKA activators may potentiate the beneficial effects of cannabinoids against glutamate-induced toxicity, as well as reduce the effect of synthetic cannabinoid WIN 55,212 on the inhibition of presynaptic glutamate release [68,69], demonstrating that the effects of cannabinoids could be dependent on the specific combination of cellular context and cannabinoids under study. Additionally, although CB1 receptors regulate the activity of adenylate cyclase, cAMP production, and PKA activity, CB1 receptors are substrates for PKA phosphorylation that, in turn, may affect their activity and intracellular outcomes as well [69].

**Figure 1:**
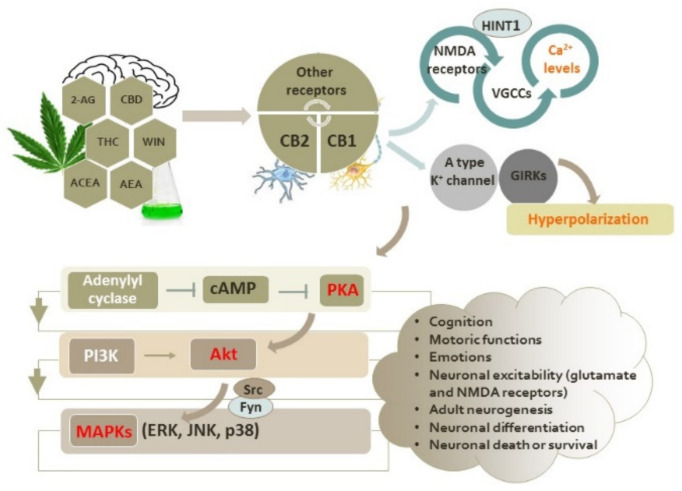
Schematic representation of signaling pathways and downstream targets mediated by cannabinoids. ACEA—arachidonoyl-2-chloroethylamide; AEA—anandamide; 2-AG—2-arachidonoyl glycerol; cAMP—cyclic AMP; CBD—cannabidiol; GIRKs—G protein-coupled inwardly rectifying potassium channels; PI3K—phosphoinositide 3-kinase; PKA—protein kinase A; MAPKs—mitogen-activated protein kinases; THC—Δ^9^-tetrahydrocannabinol; VGCCs—voltage-gated calcium channels; WIN—WIN 55,212-2.

In addition to PKA, CB1 receptors may modulate the activity of other kinases, as well as the activity of β-arrestin 1 and 2 that are involved in receptor internalization, recycling and degradation [10,70,71]. The activation of CB1 receptors is highly implicated in the regulation of mitogen-activated protein kinases (MAPKs) and the phosphoinositide 3-kinase (PI3K)/Akt pathway, which have important roles in determining neuronal death or survival, particularly in oxidative stress conditions (Figure 1) [72,73,74,75,76,77]. The release of βγ-subunits from G_i_ and G_O_ proteins is known to stimulate small G proteins such as Ras that further may lead to the activation of ERK and JNK cascades via the PI3K cascade [78]. In N1E-115 neuroblastoma cells, the synthetic cannabinoid receptor agonist WIN 55,212-2 activates the ERK pathway following PKA inhibition via CB1 receptors, but the basal activity of PI3K and the Src kinase and protein phosphatases are required for ERK activation, probably to prime the pathway and maintain viability [79]. On the contrary, the activation of ERK via CB1 receptors in the hippocampus was found to be insensitive to PI3K inhibition [80]. The activation of the ERK1/2 pathway following treatment with phytocannabinoids and synthetic cannabinoids that act as agonists of CB1 receptors was shown to have a good correlation with ability of cannabinoids to inhibit adenylyl cyclase [81]. However, in CHO cells with the overexpression of CB1 receptors, ERK1/2 activation was shown to be independent of cAMP metabolism, indicating that CB1 activation may independently trigger the adenylate cyclase and ERK1/2 pathways [63]. Later, it was found that CB1 receptor agonist methanandamide, a synthetic analogue of anandamide that is highly resistant to hydrolysis, induced a biphasic and sustained ERK1/2 response in primary neurons obtained from a 7-day-old embryonic chick telencephalon. At first, ERK1/2 activation is mediated by the sequential activation of G_q_, PLC, and Ca^2+^-independent PKCε (the activation of PLC results in phosphatidylinositol 4,5-bisphosphate hydrolysis, DAG (and IP_3_) formation, and PKC activation upstream of ERK). PKCε, which is associated with the CB1 receptors in basal conditions, dissociates from the CB1 receptors upon activation, forms a transient signaling complex with the Src and Fyn tyrosine kinases, and elicits the first round of ERK1/2 phosphorylation. The second wave of ERK1/2 activation is mediated by PTX-sensitive G_i/o_ signaling, whereby additional Fyn and Src molecules serve as effectors [82]. In stably transfected CHO cells, the activation of two major isoforms of JNK, JNK-1 and JNK-2, was shown to be induced by several endocannabinoids, phytocannabinoids, and synthetic cannabinoids and to be blocked by PTX, PI3K inhibitor wortmannin, and a Ras farnesyltransferase inhibitor peptide, indicating the contribution of CB1 receptors and the PI3K/Akt and ERK pathways in JNK stimulation. Likewise, the activation of the JNK pathway was demonstrated in neuronal cells with the endogenous expression of CB1 receptors [78]. However, endogenous cannabinoids may additionally activate the JNK pathway in a CB1 receptor-independent manner [78]. Furthermore, the activation of the p38 pathway was observed in cells treated with Δ^9^-tetrahydrocannabinol (THC), the major psychoactive component of marijuana [78].

The CB1 receptor-elicited activation of the PI3K/Akt pathway is often involved in the neuroprotective effects of cannabinoids [83,84]. Antiapoptotic and neuroprotective effects mediated by the PI3K/Akt pathway were also demonstrated following the activation of CB2 receptors [85]. The protective effects of cannabinoids have also been observed in astrocytes where cannabinoids prevented ceramide-induced apoptotic death by activating the PI3K/Akt pathway [86]. Briefly, ceramides are sphingosine-based lipids that act as signaling molecules, and their proapoptotic activities are partially induced by the ability to inhibit the Akt pathway [87]. However, CB1 receptors and cannabinoids may modulate the metabolizing pathways of sphingolipids by inducing sphingomyelin breakdown and by increasing ceramide levels via enhanced de novo synthesis [88]. Hence, the interplay between the beneficial effects of cannabinoids and their ability to increase the level of pro-apoptotic molecules such as ceramides needs to be investigated in future studies. Additionally, cannabinoids were found to protect serum-deprived astrocytes from H_2_O_2_-induced oxidative damage and death through a mechanism dependent on CB1 receptors [89].

Besides activating intracellular transduction pathways, the other major activity of cannabinoids via the CB1 receptors is related to ion channels. Thus, the stimulation of CB1 receptors may activate A-type potassium channels and inwardly rectify K^+^ currents via the G protein-coupled inwardly rectifying potassium channels (GIRKs), resulting in membrane hyperpolarization [45,90]. Hence, the activation of CB1 receptors reduces neuronal excitability, which may be important for the treatment of epilepsy [91]. Furthermore, cannabinoids potently inhibit presynaptically located, excitatory N- and P/Q-type voltage-dependent calcium channels and reduce the postsynaptic glutamate-induced accumulation of Ca^2+^ ions [45,68].

### 2.1. Interactions of Cannabinoid Receptors with Other GPCRs

CB1 receptors may interact with other receptors, mainly with other GPCRs, usually inducing alterations in the activities of both receptors. The interactions between CB1 receptors and dopamine type 2 (D2) receptors have been demonstrated in striatal neurons. The net result of that interplay is the preference of a receptor complex for signaling through the otherwise nonpreferred G_α_ protein pathway and a consequent cAMP increase [92]. In another study, a D2 receptor agonist quinpirole regulated the transcription of the *CNR1* gene and increased the mRNA levels of the CB1 receptor. In particular, the activation of the short-form D2 receptor (D_2S_R) enhanced the promoter activity of the *CNR1* gene in an ERK1/2-dependent manner [93]. Functional heteromeric complexes are also formed between the adenosine A2A receptors and CB1 receptors in rat striata, and they mediate the effects of cannabinoids on motor activities. The inhibition of A2A receptors attenuated the motor depressant effects induced by the administration of cannabinoids, indicating that the activation of A2A receptors could be a prerequisite for CB1 receptor signaling [40]. The simultaneous coactivation of CB1 receptors and μ opioid receptors was found to attenuate CB1 receptor signaling (as well as μ-opioid receptor signaling) in neuroblastoma SK-N-SH cells and striatal tissue. The physiological relevance of this μ–CB1 antagonistic crosstalk was investigated in Neuro2A cells. Coactivation resulted in substantial decreases in Src and STAT3 phosphorylation and CB1 receptor-mediated neuritogenesis in comparison with individual CB1 receptor activation [12]. A reciprocal inhibition between CB1 receptors and GABA_B_ receptors was observed in hippocampal cells [94]. Interestingly, in several regions of the rat brain and CB2 receptor-transfected neuroblastoma SH-SY5Y cells, CB1 receptors were shown to form heteromers with the CB2 receptors. A bidirectional cross-antagonism is a specific characteristic of the CB1–CB2 receptor interaction, meaning that the antagonist of one receptor blocks the effect of the other receptor agonist in the CB1–CB2 receptor heteromer and inhibits the function of the entire complex [95]. Through this interaction, CB2 receptors negatively modulate the function of CB1 receptors. For example, in neuroblastoma cells, CB2 receptors were shown to inhibit the Akt phosphorylation and neurite outgrowth induced by a CB1 receptor agonist, whereas in globus pallidus, a specific CB2 receptor antagonist blocked the activation of the ERK1/2 pathway induced by a CB1 receptor agonist [95].

CB1 receptors are also present in neuronal mitochondria, implying their contribution to energy metabolism [96,97]. As the impairment of mitochondrial functions is one of the most important consequences of oxidative stress that further amplifies ROS production and triggers apoptotic pathways, the preservation of mitochondrial functions is appreciated as an important target in neuroprotection [98,99]. The activation of mitochondrial receptors by synthetic cannabinoid arachidonoyl-2-chloroethylamide (ACEA) was shown to improve mitochondrial functions and to reduce the oxidative stress and initiation of the apoptotic cascade [100]. However, two possible outcomes have been observed in the model of traumatic brain injury upon the upregulation of mitochondrial CB1 receptors. By inhibiting mitochondrial cAMP/PKA/complex I, mitochondrial CB1 receptors were shown to exacerbate energy depletion and neuronal apoptosis, whereas the coactivation of mitochondrial Akt/complex V was shown to attenuate metabolic deficits and prevent apoptotic events [84].

Finally, it should be mentioned that endogenous and exogenous cannabinoids preferentially activate distinct signaling pathways over others, a phenomenon called biased signaling. For example, 2-AG showed little preference for the inhibition of cAMP production and the activation of the ERK1/2 pathway, whereas anandamide was seven times more biased toward cAMP inhibition [101]. The same trend was observed for phytocannabinoids and synthetic cannabinoids and was attributed to a selection bias for either G protein- or β arrestin-mediated signaling, depending on the cell type and pathophysiological conditions [9,102]. It is proposed that cannabinoids display multiple modes of action and biased signaling due to the presence of various allosteric sites and the wormhole-like structure of the orthosteric site. Different modes of cannabinoid binding probably induce distinct receptor conformations and may ensure functional selectivity [55].

### 2.2. Functional Interplay between the Cannabinoid CB1 and CB2 Receptors and Glutamate NMDA Receptors

Related to neurodegenerative pathology, it is important that the activation of the ECS controls the glutamate NMDA receptors whose overactivation and consequent excessive increase in intracellular calcium levels may initiate a cascade of events leading to neuronal death. This course of biochemical changes is termed excitotoxicity and largely contributes to the pathological mechanisms in neurodegenerative diseases [73,76,103]. The activation of NMDA receptors and increased Ca^2+^ levels upregulate the activity of numerous enzymes, including inducible nitric oxide synthase (iNOS), phospholipases, COXs and some proteolytic enzymes such as calpains. The NO produced by iNOS reacts with superoxide anion, generating extremely dangerous peroxynitrite and other ROS moieties. By inducing lipid peroxidation, mitochondrial damage, and the depletion of glutathione (GSH) levels, the excessive amounts of peroxynitrite ultimately reduce the antioxidative capacity of neuronal cells and exacerbate oxidative stress, induce the oxidative damage of cellular proteins via tyrosine nitration, and deregulate redox-sensitive signaling pathways and energy production, thus leading to the severe impairment of neuronal functioning and, ultimately, neuronal death [8,38,62,104].

The increased activity of NOS and the overproduction of ROS, as well as the enhanced nitration of CB1 and CB2 proteins, have been observed in Alzheimer’s disease (AD), suggesting that peroxynitrite-mediated oxidative damage is an important contributing factor to disease progression [105,106,107]. The pathology of Parkinson’s disease (PD) is also related to the imbalance of peroxynitrite formation. The peroxynitrite-mediated nitration of α-synuclein promotes its aggregation, a typical hallmark of PD pathology. Afterwards, peroxynitrite contributes to the nitration of tyrosine hydroxylase, which is essential for dopamine synthesis. As distinct tyrosine residues determine the substrate specificity of monoamine oxidase B (MAO B), this may further deregulate dopamine metabolism. Additionally, peroxynitrite inactivates glutathione reductase, depleting the intracellular GSH content in substantia nigra [8,108,109]. In a rotenone-induced animal model of PD, a natural CB2 receptor agonist β-caryophyllene demonstrated neuroprotective effects. Via CB2 receptors, it reduced the loss of the activity of the superoxide dismutase (SOD) and catalase antioxidant enzymes, reduced malondialdehyde (MDA) levels, restored GSH content, prevented an increase in nitrite levels, and alleviated glial activation and the induction of the proinflammatory cytokines IL-1β, IL-6, and tumor necrosis factor alpha (TNF-α) and the inflammatory mediators NF-κB, COX-2, and iNOS [110]. Likewise, oxidative stress and the increased production of NO and peroxynitrite are important contributing factors to Huntington’s disease (HD) [111].

Several lines of evidence indicate that CB1 receptor agonists protect neurons from the toxic effects induced by excess glutamate exposure [11,83,112,113]. In cortical neurons, it was shown that the neuroprotective effects of cannabinoids against glutamate-induced neurotoxicity are dependent on enhanced cAMP levels and that cannabinoid receptors reduce excitotoxicity only when they are concomitantly activated with glutamate exposure [68]. As cannabinoids also inhibit voltage-sensitive calcium channels (which mediate approximately half of the Ca^2+^ entry during exogenous glutamate exposure), it is likely that the neuroprotective effects of cannabinoids are based on a reduced influx of Ca^2+^ ions during excitotoxicity [68]. The main cannabinoid-elicited mechanisms involved in the regulation of NMDA receptor function and the prevention of their overactivation include the reduction in presynaptic glutamate release, the inhibition of the post-synaptic cannabinoid receptors that modulate NMDA receptors and NMDA-regulated signaling cascades, and the direct inhibition of NMDA channels and Ca^2+^ influx [8,36,38,114]. In striatal neurons, the activation of CB1 receptors was found to prevent NMDA-induced excitotoxic death via the PI3K/Akt/mTOR pathway and to stimulate the expression of brain-derived neurotrophic factor (BDNF) [83]. In another study in which the genetic or pharmacological inhibition of CB1 receptors resulted in the increased neurotoxic effects of kainic acid in hippocampal cultures, it was similarly shown that BDNF is an important mediator of CB1 receptor-mediated neuroprotection [115].

CB1 receptors associate with NMDA receptors via the NR1 subunits of the NMDA receptors. The HINT1 scaffold protein is important for the stabilization of the CB1–NR1 interaction. When CB1 receptors are coupled to NR1 subunits via HINT1, cannabinoids stimulate the co-internalization of the entire heteromer, protecting the neuronal cells from NO production and excitotoxicity by reducing the number of surface NMDA receptors [38,116]. However, it seems that CB1 agonists differ in their ability to sequester CB1 receptors. Agonists that promote the strong internalization of CB1 receptors and simultaneously provide effective neuroprotection were found to be synthetic cannabinoids such as WIN 55,212-2 and ACEA, whereas endocannabinoid anandamide was found to be much less efficient in removing CB1 receptors from the cell surface [38]. In another study, Kreutz et al. [117] investigated the neuroprotective effects of THC, anandamide, and 2-AG in organotypic hippocampal slice cultures exposed to NMDA. All three cannabinoids reduced microglial activation, but only 2-AG reduced the number of degenerating neurons. Later, the authors proposed that 2-AG exerts it effects by activating non-CB1/non-CB2 cannabidiol-sensitive receptors present on microglial cells [118]. However, besides decreasing the levels of Ca^2+^ ions, the activation of CB1 receptors may enhance the NMDA-induced increase in Ca^2+^ release from the inositol-triphosphate (IP_3_)-sensitive intracellular stores, further indicating that the cellular mechanisms of cannabinoid actions are rather complex and still not fully defined [119]. NMDA and CB2 receptors also form functional complexes that alter the effects exerted by CB2 or NMDA agonists. In HEK-293T cells expressing the NR1 subunit, NR2 subunit, and CB2 receptors, both the cAMP decrease and ERK activation induced by JWH-133 (a selective agonist of CB2 receptors) were shown to be counteracted by the NMDA receptor activation (negative crosstalk). More importantly, JWH-133 was shown to inhibit NMDA-induced calcium influx, indicating that cannabinoids may restrain NMDA receptor activation at the CB2–NMDA complex. In hippocampal neurons from APP_Sw/Ind_ transgenic mice, the CB2–NMDA heteromer was found to be highly overexpressed, but cross-antagonism was not observed [55].

## 3. Endocannabinoid System in Neurodegenerative Diseases

The altered expression of endocannabinoids and cannabinoid receptors has been observed in neurodegenerative conditions (Table 1). Accordingly, it has been assumed that endocannabinoid-degradative enzymes, CB1 and CB2 receptors, and the modulation of the activity of endogenous cannabinoids represent valuable therapeutic targets in neurodegenerative diseases, as well as in other diseases such as epilepsy, stroke, inflammation, multiple sclerosis, traumatic brain injuries and psychiatric illnesses [4,120,121,122,123].

In HD, a reduced density of CB1 receptors has been found before the onset of the first symptoms in disease mutation carriers and in symptomatic HD patients, whereby the number of CAG repeats in the *HTT* gene was negatively correlated with the CB1 receptor density in the prefrontal and premotor cortices [83,124,125,126]. In the R6/2 mouse, a well-established model of HD, the re-expression of the CB1 receptors by adeno-associated viral vector normalized otherwise reduced levels of BDNF in the dorsolateral striatum and rescued striatal atrophy [83]. However, Sativex, a botanical extract with equimolar amounts of THC and cannabidiol (CBD) (the two major active ingredients of marijuana) did not improve motor, cognitive and behavioral deficits or induce molecular changes in HD patients [127]. On the other hand, agonists of CB2 receptors, but not agonists of CB1 receptors, were found to protect striatal projection neurons from death in a rat model of HD induced by the intrastriatal injection of malonate, an inhibitor of the mitochondrial complex II that induces apoptosis and microglial activation. The increased expression of CB2 receptors in astrocytes and reactive microglia was observed during the progression of striatal degeneration, and CB2 receptor agonists reduced the production of TNF-α and gliosis but did not affect the mechanisms of antioxidative defense such as the expression of SOD-1 and SOD-2, altogether suggesting that targeting CB2 receptors is a promising approach against neuronal injury in diseases that are accompanied by the upregulation of CB2 receptors in glial cells [54]. On the other hand, in one study, THC exacerbated neurodegenerative changes induced by the intrastriatal administration of malonate. Surprisingly, an even more pronounced effect on malonate-induced striatal lesions was observed for SR141716, a selective CB1 antagonist, suggesting that the activation of CB1 receptors produces neuroprotective effects [128]. Importantly, in a cellular model of HD, biased signaling properties have been observed. Endocannabinoids 2-AG and anandamide displayed preference to Gα_i/o_-dependent ERK phosphorylation that normalized the levels of CB1 receptors and improved the viability of HD cells, whereas THC preferentially activated β-arrestin 1 recruitment, further depleting the levels of CB1 receptors and cell survival. This study suggests that the enhancement of Gαi/o-biased endocannabinoid signaling is a reliable pharmacological approach in HD that should be exploited to limit the adverse on-target effects of potent synthetic cannabinoids [102]. Functional selectivity at the CB2 receptors was also demonstrated [129].

Regarding AD, the reduced expression of CB1 receptors has been observed in AD brains, particularly in areas of microglial activation [105]. Alterations of CB1 receptor expression are regionally specific and dependent on the course of the disease [130]. One study showed that the overall CB1 receptor levels were unchanged in the hippocampi of AD patients, but the protein levels of the enzymes involved in the synthesis and degradation of endocannabinoids were altered: sn-1-DAGL α and β isoforms, enzymes synthesizing 2-AG, were significantly increased in Braak stage VI, serine hydrolase α/β-hydrolase domain-containing 6 expression disappeared in neurofibrillary tangle-bearing neurons, and MAGL expression was reduced in comparison with pyramidal cells without signs of neurofibrillary pathology [131]. The activity of FAAH was also reduced in the frontal cortices of AD patients [132], together with depleted levels of anandamide and its precursor 1-stearoyl, 2-docosahexaenoyl-sn-glycero-phosphoethanolamine-N-arachidonoyl in the midfrontal and temporal cortices of AD. Moreover, the levels of anandamide and its precursor were positively correlated with cognitive deficits and inversely correlated with Aβ levels [133]. In another study, the expression of CB1 receptors was reduced in post mortem cortical brain tissue but did not correlate with cognitive status and the molecular markers of the disease. However, in the same study, an increase in the expression CB2 receptors was positively correlated with the Aβ42 levels and senile plaque score [134]. Moreover, CB2 receptor agonists were efficient in promoting Aβ clearance and the reversal of cognitive deficits. They also attenuated microglial activation and the production of interleukin (IL)-1β, and they prevented the upregulation of CB2 receptors [135]. Interestingly, in an animal model of AD with CB1 receptor deficiency (obtained by breeding amyloid precursor protein (APP) Swedish mutant mice (APP23) with CB1-deficient mice), more pronounced learning impairments and memory deficits were observed together with the reduced plaque deposition [136]. AD pathology was also shown to be accompanied by increased levels of 2-AG in the plasma of AD patients [137] and the hippocampi of rodents administered with the Aβ42 peptide [138]. As VDM-11, an inhibitor of endocannabinoid cellular uptake, reverses hippocampal damage and cognitive deficits when concomitantly applied with Aβ42 at the early stages of Aβ42 treatment, it seems that an early increase in endocannabinoids serves a protective role against Aβ toxicity [138]. Increases in 2-AG may also affect the immune response and pathological hallmarks of AD. It has been shown that MAGL inhibitors (which increase 2-AG levels) reduce the proinflammatory response of microglia and astrocytes, the expression and activity of β-secretase-1 (BACE1), and the Aβ burden in the hippocampus and the temporal and parietal cortices, as well as improve cognitive impairments, in animal models of AD [139,140]. Cannabinoid-profiled compounds (endocannabinoids, FAAH inhibitors, and synthetic cannabinoids) have also demonstrated neuroprotective effects in combined high glucose and Aβ conditions [141]. They improved the viability of primary hippocampal neurons; reduced the aggregation of Aβ, ROS formation and nitrosative stress; modified the enzymatic activity of SOD, catalase and antioxidant enzymes involved in glutathione homeostasis; reduced the formation of end products of lipid peroxidation and the levels of inflammatory markers (iNOS, IL-1β, and TNF-α); prevented decreases in mitochondrial membrane potential; and stimulated Nrf2 and CREB phosphorylation. At least partially, the protective effects of anandamide and synthetic cannabinoid WIN 55,212-2 were achieved via its direct scavenging ability [141].

Elevated levels of anandamide and CB1 receptors were found in the basal ganglia and cerebrospinal fluid of patients with PD, probably as a compensatory mechanism to counteract dopamine depletion. Of note, anandamide levels were restored in patients under chronic dopaminergic therapy [142,143], although clinical results with CB1/CB2 receptor agonists failed to show encouraging results regarding motor disabilities [9]. However, cannabis smoking improved motor symptoms in one study, suggesting that several marijuana components with synergistic activity could be a better approach than treatment with individual cannabinoids in alleviating the motor symptoms of PD patients [144]. The activation of CB2 receptors also demonstrated great potential in PD by attenuating the inflammatory response [145]. In a 1-methyl-4-phenyl-1,2,3,6-tetrahydropyridine (MPTP)-induced model of PD, the activation of CB2 receptors prevented the degeneration of dopaminergic neurons in substantia nigra by reducing the damage of the blood–brain barrier, the infiltration of peripheral immune cells, and the expression of iNOS and pro-inflammatory cytokines after microglial activation [145].

In a transgenic G93A-SOD1 mice model of amyotrophic lateral sclerosis (ALS), increased levels of anandamide and 2-AG were found in the spinal cord, probably as a mechanism of endogenous defense as changes were observed before overt motor deficits [146]. In the human spinal cords of ALS patients, the upregulation of CB2 immunostaining was also observed post mortem, probably reflecting the activation of microglial cells [147].

**Table 1:** Endocannabinoid system (ECS) in neurodegenerative diseases.

Disease	ECS	Observed Change	Model	Reference
HD	Receptors	↓ CB1R	R6/2 mouse	[83]
		↓ CB1R	pre-HD mutation carriers and symptomatic HD patients	[124]
		↓ CB1R	basal ganglia of patients	[126]
		↓ CB1R	grey matter of patients	[127]
		↑ CB2R in astrocytes and reactive microglia	malonate-induced rat model	[54]
AD	EC	↓ anandamide and its precursor	midfrontal and temporal cortices of patients	[133]
		↑ 2-AG	plasma of patients	[137]
		↑ 2-AG	hippocampi of rat model	[138]
	Receptors	↓ CB1R	brains of AD patients	[105]
		alterations of CB1R expression	mouse model of AD	[130]
		unchanged levels of CB1R	hippocampi of patients	[131]
		↓ CB1R	post mortem cortical brain	[134]
		CB1R deficiency	rat model of AD	[135]
	EC enzymes	↑ sn-1-DAGL α and β isoforms, no expression of ABHD6, ↓ MAGL	hippocampi of patients	[131]
		↓ FAAH	frontal cortices of patients	[132]
PD	EC	↑ anandamide	cerebrospinal fluid of patients	[143]
	Receptors	↑ CB1R	basal ganglia of patients	[142]
ALS	EC	↑ anandamide and 2-AG	spinal cords of SOD1 G93A mice	[146]
	Receptors	↑ CB2R	human spinal cord	[147]

↓ decreased level, expression or activity; ↑, increased level, expression or activity; ABHD6, α/β-hydrolase domain-containing 6; AD, Alzheimer’s disease; ALS, amyotrophic lateral sclerosis; 2-AG, 2-arachidonoylglycerol; CB1R, cannabinoid receptor type 1; CBR2, cannabinoid receptor type 2; DAGL, DAG lipase; EC, endocannabinoids; ECS, endocannabinoid system; FAAH, fatty acid amide hydrolase; HD, Huntington’s disease; MAGL, monoacylglycerol lipase; PD, Parkinson’s disease.

The involvement of the ECS has also been recognized in epilepsy, as changes in the expression of CB1 receptors and endocannabinoids have been detected in some patients. For example, depleted levels of anandamide, but not 2-AG, were found in the cerebrospinal fluid of untreated patients with temporal lobe epilepsy [148], whereas increased CB1 receptor signaling efficacy (despite the low mRNA and protein expression), increased levels of anandamide, and low 2-AG levels were noticed in the hippocampi of patients with pharmacoresistant temporal lobe epilepsy [149]. Yet another study revealed the downregulation of the CB1 receptor mRNA, the decreased expression of DAGL-α, and the reduced fraction of CB1-positive glutamatergic nerve endings [150]. Several lines of evidence indicate the anticonvulsive potential of cannabinoids [1,4,151], although caution is required as the acute administration of the synthetic cannabinoid AM2201 induced epileptic seizures in freely moving mice. Seizures were mediated by CB1 receptors and related to the rapid elevation of hippocampal glutamate release [152].

Similarly, brain damage after cerebral ischemia was found to be more prominent in CB1 receptor knockout mice, suggesting the neuroprotective role of CB1 receptors [121], although the neuroprotective effects of CB1 receptor antagonist were also observed. In ischemic rats, the administration of CB1 receptor antagonist AM251 reduced CA1 injury and behavioral deficits [153]. CB1 and CB2 receptor antagonists were also able to prevent minocycline-mediated neuroprotective effects in traumatic brain injury, implying the complex regulatory effects of cannabinoids in neuroprotection [9,154].

## 4. Antioxidant Capacity of Cannabinoids

Briefly, oxidative stress and accompanying neuroinflammation are important underlying mechanisms of pathophysiological changes in neurodegenerative diseases. Oxidative stress develops when the production of ROS overwhelms the endogenous capabilities of the antioxidative defense provided by diverse enzymatic and non-enzymatic mechanisms. Non-enzymatic antioxidants (e.g., GSH and vitamins) and antioxidant enzymes (catalase, SOD, glutathione peroxidase, and thioredoxin) are critically involved in redox regulation and the maintenance of redox homeostasis [155,156,157,158,159,160]. In comparison with other tissues, the brain is particularly vulnerable to oxidative damage due to its high rate of oxygen consumption, limited antioxidative defense, presence of metal ions that are able to initiate redox cycling and the formation of highly dangerous and very reactive hydroxyl radicals via Fenton chemistry, and the high amount of polyunsaturated fatty acids (PUFAs) that are prone to oxidation, together with the post-mitotic nature of neurons [161,162]. Besides disturbing redox homeostasis, ROS (as highly reactive moieties) interact with cellular macromolecules (nucleic acids, proteins, lipids, and carbohydrates), impairing their structure and threatening cellular functioning. Lipids, PUFAs in particular, undergo ROS-initiated lipid peroxidation, a process of free radical formation in lipid compartments of the membrane that may ultimately disturb the permeability and fluidity of cellular membranes. Highly reactive aldehydes, such as 4-hydroxynonenal (HNE) and malondialdehyde (MDA), are typical end products of lipid peroxidation that act as signaling molecules; react with proteins and enzymes, leading to their modification and altered activity; and induce damage of cellular proteins and DNA. Their levels are increased in the diseased brain, suggesting the involvement in neurodegenerative events [163,164]. Oxidatively damaged proteins are more prone to self-aggregation, which is a characteristic hallmark of neurodegeneration. Protein aggregates induce the widespread activation of glial cells and, similarly to HNE, activate inflammatory signaling cascades. Immune activation is yet another typical hallmark of neurodegenerative diseases that maintains the sustained release of proinflammatory mediators and the production of reactive oxygen and nitrogen species. The inflammatory response is predominantly mediated by microglial cells that are part of the innate immune system. Although the primary function of the neuroinflammatory response should be protective and essential for the maintenance of brain homeostasis, prolonged and uncontrolled microglial overactivation exacerbates neuronal damage by producing a wide range of pro-inflammatory and cytotoxic molecules, such as the tumor necrosis factor (TNF)-α, interleukin (IL)-1β, IL-1, and IL-6 cytokines [155,165,166,167]. As previously mentioned, the most prominent activators of immune response are aggregated protein forms and other danger-associated molecular patterns originating from damaged and dying cells. These molecular motifs activate the nuclear factor kappa-light-chain-enhancer of activated B cells (NF-κB) transcription factor that initiates the production of inflammatory cytokines and chemokines that recruit microglial cells to the site of injury [157,168]. NF-κB also directs the expression of the superoxide anion-producing enzyme NADPH oxidase and the NO-producing enzyme iNOS. In a vicious loop of neuroinflammatory response predominantly mediated by activated microglia, the stimulation of inflammatory signaling cascades further promotes ROS production, oxidative and nitrosative stress, protein oxidation and aggregation, and mitochondrial impairment together with ATP deficiency and bioenergetics failure, the release of proinflammatory cytokines, and neuronal damage, which finally results in the impairment of neuronal functioning and ultimately neuronal death [8,169,170]. In addition to neuroinflammation, oxidative stress also induces endoplasmic reticulum (ER) stress, calcium overload, and the impairment of calcium homeostasis that, together with excitotoxicity, further contribute to the progression of neuropathological events exacerbating ROS production [171,172,173].

Cannabinoids exert direct and indirect antioxidant properties in neuronal cells. The direct antioxidant capacity of cannabinoids is dependent on the ability of their polyphenol group to transfer electrons or hydrogen atoms to oxidants [174]. There are two main proposed mechanisms of the antioxidative activity of cannabinoids: the transfer of a hydrogen atom, whereby a free radical removes a hydrogen atom from an antioxidant, and the donation of an electron from the antioxidant to the radical [175]. Moreover, it has been shown that sublethal oxidative stress, at least the one induced by *tert*-butylhydroperoxide (tBHP), increases nuclear localization and the expression of FAAH and upregulates the expression of CB1 and CB2 receptors, indicating that these changes are part of the early response to oxidative damage and potential targets for pharmacological intervention [176].

As previously mentioned, among different signaling pathways activated by oxidative stress and neuroinflammation, of particular importance are those mediated by NF-κB, which directs the synthesis of proinflammatory cytokines and induces the expression of iNOS and COX-2, and by nuclear factor-erythroid-2-like 2 (Nrf2), which regulates the expression of antioxidant mediators such as SOD1, heme oxygenase-1 (HO-1), catalase, and glutathione S-transferase. Following nuclear translocation, Nrf2 interacts with antioxidant response element (ARE) and orchestrates a neuronal response to oxidative injury by driving the transcription of antioxidant genes [157,177]

The effects of cannabinoids on NF-κB and Nrf2 signaling should be further investigated as targeting these pathways has been considered an important neuroprotective strategy with relevant therapeutic implications [177,178,179]. A putative ARE motif was found in the *CNR2* receptor gene and displays a high similarity with the consensus ARE sequence. However, one study demonstrated that in hippocampal HT22 cells and primary neurons, Nrf2 is not able to regulate the expression of CB2 receptors, whereas in microglial cells, the expression of CB2 receptors was found to be Nrf2-dependent [180]. Recently, it was shown that hexocannabitriol, a hydroxylated CBD analogue isolated from hemp threshing residues, may activate the Nrf2 pathway in a ROS-independent way, probably as a result of direct Nrf2 stabilization [181].

### 4.1. Antioxidative and Neuroprotective Effects of Endocannabinoids

Anandamide, which is the CB1 receptor ligand without a cannabinoid structure, does not have a significant oxidation potential and possesses a modest direct antioxidant ability. To investigate whether cannabinoids can protect neurons against glutamate neurotoxicity by directly reacting with ROS, Hampson et al. [182] used cyclic voltammetry to determine ability of cannabinoids to donate or accept electrons. In contrast to CBD, THC, and several synthetic cannabinoids that all readily donated electrons, anandamide did not undergo oxidation and was considered a nonresponsive compound. As several cannabinoids demonstrated a considerable antioxidative effect, it was suggested that antioxidant activity is an intrinsic property of the cannabinoid structure [182]. Despite the aforementioned findings, the neuroprotective effects of anandamide in oxidative stress conditions have been observed (Table 2). Thus, anandamide was shown to protect hippocampal HT22 cells against H_2_O_2_-induce injury via a CB1-dependent mechanism. It upregulated the expression of CB1 receptors, ameliorated H_2_O_2_-induced morphological changes, decreased the levels of cleaved caspase-3 (a measure of apoptotic rate), decreased the intracellular accumulation of ROS, restored SOD activity, and partially replenished GSH content and the GSH/GSSG ratio. As anandamide attenuated the expression of NADPH oxidase 2 (Nox2), which largely contributes to oxidative injury in the brain, and the Nox2 inhibitor apocynin exerted the same neuroprotective effect as anandamide, the authors proposed that the observed antioxidative effects of anandamide were achieved through the CB1 receptor-mediated suppression of Nox2 [183]. Anandamide also protected a perinatal brain against 2-amino-3-(4-butyl-3-hydroxyisoxazol-5-yl)propionic acid (AMPA)/kainate receptor-mediated excitotoxic lesions via CB1 receptors [184].

Due to the rapid metabolic inactivation of anandamide, its levels are relatively low in vivo, which limits its clinical application. Based on the anandamide structure, the compound N-linoleyltyrosine (NITyr) was synthesized, and its antioxidative properties were investigated in rat pheochromocytoma PC12 cells. Via CB1 receptors, NITyr attenuated H_2_O_2_-induced neurotoxic effects, reduced ROS generation, and induced autophagy by stimulating the expression of autophagy-related proteins. As the autophagy inhibitor attenuated the effects of NITyr on ROS levels and cell survival, it is likely that the observed antioxidative effects were mediated by autophagy and that the CB1 receptor-mediated induction of autophagy may be a promising neuroprotective approach in future pharmacological strategies [185]. This compound also demonstrated neuroprotective effects in primary cortical neurons. It attenuated Aβ40-induced toxicity, increased BDNF levels, and promoted autophagy by activating the CB2/AMPK/mTOR/ULK1 pathway [186]. However, anandamide may influence the cortical and cerebellar responses of NMDA receptors and calcium influx via CB1-dependent and-independent mechanisms in opposite directions [114]. When voltage-sensitive calcium channels are activated due to the depolarization induced by the activation of NMDA receptors and calcium entry, cannabinoids inhibit NMDA receptors and reduce the overall Ca^2+^ flux. On the other hand, by potentiating NMDA receptor activity (CB1-independent mechanism), anandamide may enhance calcium intake [114]. Accordingly, not all studies observed the neuroprotective effects of anandamide. For example, the effects of endocannabinoids were investigated in spinal cord injury, where excitotoxicity is the key factor of neuronal damage. Anandamide, as well as 2-AG, failed to attenuate electrophysiological and histological impairments, although the CB1 antagonist exacerbated neuronal injury, indicating that the ECS was activated but not enough to reduce the damage, at least during the early phases of the excitotoxic response to spinal cord injury [187].

*N*-oleoylethanolamine (OEA), an endocannabinoid, also demonstrated neuroprotective effects acting as a PPARα agonist. The PPARs are transcription factors that behave as lipid-sensing receptors and regulate the expression of large sets of genes predominantly involved in cellular metabolism and energy homeostasis, but they also may interfere with the inflammatory transcription factor signaling and regulate inflammatory response [188,189]. In a mouse model of cerebral ischemia, OEA reduced infarct volume, increased the expression of the inhibitory protein IκBα of the NF-κB signaling pathway, and reduced the expression of the inflammatory marker COX-2 [13,190]. In closed head injuries, the neuroprotective effects of exogenously added 2-AG were also found to be mediated through the NF-κB activation via a CB1 receptor-mediated mechanism and the suppression of the mRNA expression of proinflammatory cytokines [120,191,192].

The inhibition of enzymes involved in cannabinoid synthesis has also been shown to exert neuroprotective effects. The inhibition of MAGL, the 2-AG-metabolizing enzyme that increases 2-AG levels, promoted recovery in a mouse model of repetitive mild closed head injury [193]. The MAGL inhibitor also reduced the expression of proinflammatory markers IL-1β, IL-6, and TNFα; attenuated microglial and astrocytic activation; reduced the formation of Aβ and the expression of enzymes that participate in Aβ synthesis in the cortex and hippocampus; inhibited tau phosphorylation and the aggregation of TDP-43; and prevented changes in the expression of the AMPA and NMDA receptor subunits [193]. In an animal model of AD, the inhibition of MAGL activity suppressed the expression and activity of BACE1 and the accumulation of Aβ, reduced inflammation and neurodegeneration, and improved long-term synaptic plasticity and cognitive abilities [139]. In SH-SY5Y cells exposed to 1-methyl-4-phenylpyridinium iodide (MPP^+^) toxicity, the MAGL inhibitor also displayed neuroprotective effects that were blocked by the CB2 receptor antagonist and mimicked by the CB2 receptor agonist [194]. In mice with a traumatic brain injury, the FAAH inhibitor enhanced anandamide levels, increased the expression of Bcl-2 and reduced neurodegeneration, improved motor and cognitive deficits, suppressed the formation of APP and the expression of iNOS and COX-2, and promoted the polarization of microglia towards a neuroprotective M2 phenotype. The effects were mediated by the CB1 and CB2 receptors and the Akt and ERK1/2 signaling pathways [195]. FAAH deletion in G93A-SOD1 mice delayed signs of the disease [196] and exerted beneficial effects on the motor symptoms in MPTP-induced toxicity [197].

**Table 2:** Antioxidant and anti-inflammatory effects of endocannabinoids in neurodegenerative conditions.

Compound	Signaling	Effects	Model	Ref.
AEA	↑ CB1R, ↓ cleaved caspase-3, ROS and Nox2, restored SOD, partially replenished GSH content and GSH/GSSG ratio	protection from H_2_O_2_-induced injury	hippocampal HT22 cells	[183]
AEA	Via CB1R	protection from AMPA/kainate receptor-induced lesions	perinatal rodent brain	[184]
AEA and 2-AG	-	failed to attenuate kainate-mediated excitotoxicity	neonatal rat spinal cord in vitro	[187]
OEA	PPARα agonist; ↑ IκBα, and ↓ COX-2	neuroprotection, reduced infarct volume	cerebral ischemia mouse model	[190]
2-AG	↓ TNF-α, IL-1β and IL-6 mRNA; ↑ endogenous antioxidants	decreased BBB permeability	closed head injury mouse model	[120]
2-AG	Via CB1R	reduction in brain edema, better clinical recovery, reduced infarct volume	closed head injury mouse model	[191,192]
MAGL inhibitor (↑ 2-AG)	↓ inflammatory markers TNF-α, IL-1β and IL-6, ↓ Aβ formation and enzymes involved in Aβ synthesis, ↓ tau phosphorylation and aggregation of TDP-43, prevented changes in the expression of AMPA and NMDA receptor subunits	↓ microglial and astrocytic activation, promoted recovery	repetitive mild closed head injury mouse model	[193]
MAGL inhibitor (↑ 2-AG)	↓ BACE1 and accumulation of Aβ, ↓ inflammation	improved synaptic plasticity and cognitive abilities, reduced neurodegeneration	mouse model of AD	[139]
MAGL inhibitor (↑ 2-AG)	Via CB2R	neuroprotection from MPP^+^-induced toxicity	SH-SY5Y cells	[194]
FAAH inhibitor (↑ AEA)	↑ Bcl-2, ↓ APP and iNOS and COX-2, via CB1R and CB2R, Akt and ERK1/2 pathways	improved motor and cognitive deficits, promoted polarization of M2 microglia, reduced neurodegeneration	traumatic brain injury mouse model	[195]
FAAH deletion (↑ AEA)	-	delayed signs of the disease	mouse model of ALS	[196]
FAAH inhibitors (↑ AEA)	Via CB1R and CB2R	prevented motor impairment, did not prevent dopamine loss, showed anti-cataleptic properties	rat model of PD	[197]

↓, decreased level, expression or activity; ↑, increased level, expression or activity; Aβ, amyloid beta; AD, Alzheimer’s disease; AEA, anandamide; ALS, amyotrophic lateral sclerosis; AMPA, 2-amino-3-(4-butyl-3-hydroxyisoxazol-5-yl)propionic acid; APP, amyloid precursor protein; 2-AG, 2-arachidonoylglycerol; BACE, β-secretase; BBB, blood–brain barrier; CB1R, cannabinoid receptor type 1; CBR2, cannabinoid receptor type 2; COX-2, cyclooxygenase-2; FAAH, fatty acid amide hydrolase; GSH, glutathione; GSSG, Glutathione disulfide; HD, Huntington’s disease; IκBα, nuclear factor of kappa light polypeptide gene enhancer in B-cells inhibitor alpha; IL-1β, interleukin-1β; IL-6, interleukin 6; iNOS, inducible nitric oxide synthase; MAGL, monoacylglycerol lipase; NMDA, N-methyl-D-aspartate; OEA, *N*-oleoylethanolamine; PD, Parkinson’s disease; PPAR, peroxisome proliferator-activated receptor; ROS, reactive oxygen species; SOD, superoxide dismutase; TDP-43, TAR DNA-binding protein 43; TNF-α, tumor necrosis factor alpha.

### 4.2. Antioxidative and Neuroprotective Effects of Phytocannabinoids

Cannabidiol (CBD) and Δ^9^-tetrahydrocannabinol (THC) are the most studied plant-derived cannabinoids. Cannabidiol (CBD) is the most abundant non-psychoactive cannabinoid from marijuana extracts, whereas THC is the major psychoactive compound. CBD displays various pharmacological effects and a relatively favorable safety profile at a variety of doses that have greatly contributed to interest in its neuroprotective properties [198,199] (Table 3). It displays a low affinity for CB1 and CB2 receptors, acts as an antagonist of the GPR55 receptor and PPAR agonist, interacts with TRPV channels, and inhibits voltage-gated calcium channels [10,200]. It also increases the endogenous levels of anandamide by inhibiting its reuptake and degradation by FAAH [200,201]. In addition, CBD stimulates synaptic plasticity and neurogenesis, which probably underlies its positive effects in depression and anxiety [202]. At least in mice, the neurogenic effect of CBD in the dentate gyrus is mediated by CB1 receptors and affects the proliferation of progenitor cells and the maturation and survival of new neurons [43]. On the contrary, prolonged THC administration was found to reduce the proliferation of precursor cells in the dentate gyrus but did not affect cell survival or net neurogenesis [43]. CBD also upregulates BDNF and reduces microglial activation and the release of proinflammatory molecules [203], all of which may contribute to the desired beneficial effects of CBD in neuroprotection.

The psychoactive effects of THC are due to the activation of CB1 receptors, although it binds with a high affinity to both CB1 and CB2 receptors [10]. A meta-analysis revealed that THC demonstrates neuromodulatory effects in the brain regions involved in cognitive tasks. Greater effects were achieved in regions with higher densities of CB1 receptors, though a relationship between *CNR2* gene expression and the effect size was not found [204]. In contrast to the psychoactive effects that are mediated by CB1 receptors, the immunomodulatory effects of THC are mediated by binding to the CB2 receptors in microglial cells [205].

The therapeutic applications of THC are limited by its side effects including the development of tolerance and addiction, along with cardiac side effects. It is interesting that growing plants contain high levels of the non-psychoactive and pharmacologically active Δ^9^-tetrahydrocannabinolic acid (THCA) that is decarboxylated into the psychoactive form of THC during heating. As THCA exerts neuroprotective and anti-inflammatory effects, this compound is worth consideration for the treatment of neurodegenerative diseases in future studies [206,207].

In addition to cyclic voltammetry, which has demonstrated the similar antioxidant potential of CBD and THC to the powerful antioxidant butylated hydroxytoluene (BHT), the abilities of CBD, THC and BHT to be oxidized were also examined in a Fenton reaction. All three tested compounds prevented dihydrorhodamine oxidation with a similar potential, indicating the remarkable antioxidant potency of CBD and THC [182]. To confirm that CBD acts as an antioxidant, primary cortical neurons were exposed to the oxidant *tert*-butyl hydroperoxide. As CBD was able to prevent the release of lactate dehydrogenase (LDH), an indicator of cellular damage, the authors concluded that CBD protected neurons from ROS-induced cell death. They also demonstrated the neuroprotective effects of CBD against glutamate-induced toxicity [182]. By comparing some specific structural and electronic characteristic of THC and CBD, such as ionization potential, the energies of the highest occupied molecular orbital and the lowest unoccupied molecular orbital, hydroxyl bond dissociation energy, and spin density, it was assessed that THC possesses better antioxidant properties than CBD due to its better electron donating ability and higher nucleophilicity [175]. It is suggested that the antioxidant capacity of THC is mostly determined by the stability of its semiquinone radical after hydrogen abstraction [174,175]. Due to the stability of its semiquinone radical after scavenging free radicals, THC is regarded as an important antioxidant and a lipoperoxidation chain-breaking agent during the propagation and termination phases of lipid oxidation, respectively [174].

Some studies have indicated that the antioxidant activity of THC and CBD per se is sufficient to protect neurons against oxidative injury without the activation of cannabinoid receptors. Both THC and CBD demonstrated protective effects in rat cortical neuronal cultures exposed to toxic levels of glutamate, and they also prevented neuronal death. They protected neurons against toxicity mediated by NMDA, AMPA, and kainate receptors. As antagonists of CB1 receptors did not abolish the neuroprotective effects of both CBD and THC, it was concluded that neuroprotective action is mediated by the direct antioxidative activity of cannabinoids [182]. CBD also prevented the lipid peroxidation induced by *tert*-butyl hydroperoxide with a better efficacy then some other antioxidants, further suggesting that direct antioxidative action is the major mechanism of its neuroprotective efficacy [182]. The effects of CBD were also investigated on oligodendrocyte progenitor cells. CBD protected these cells from H_2_O_2_-induced death and oxidative stress by reducing the levels of ROS and from lipopolysaccharide (LPS)/IFN*γ*-induced cytotoxicity by attenuating caspase-3 cleavage and apoptosis via mechanisms that were independent of CB1, CB2, TRPV1 or PPAR*γ* receptors, probably suggesting the direct antioxidant and anti-inflammatory activity of CBD. Furthermore, CBD protected oligodendrocyte progenitor cells against tunicamycin-induced ER stress (by decreasing the phosphorylation of the eiF2*α* protein) and attenuated the ER stress response during neuroinflammation. Hence, it is suggested that the prevention of the ER stress pathway underlies the beneficial effects of CBD on oligodendrocyte progenitor cells during neuroinflammation [208].

However, in addition to their direct antioxidative abilities, the neuroprotective effects of phytocannabinoids are also mediated by cannabinoid (and other) receptors. Thus, CBD was shown to protect an immature brain from hypoxic–ischemic damage. It reduced levels of glutamate and IL-6 and the expression of TNF-α, iNOS and COX-2 acting at CB2 and adenosine receptors [209]. Following exposure to TNF-α, which increases the surface expression of AMPA receptors and potentiates AMPA receptor-mediated glutamatergic excitotoxicity, the activation of CB1 receptors reduced the number of AMPA receptors and mitigated TNF-α-induced excitotoxic death in hippocampal neurons. Among other investigated cannabinoids, this effect was observed following exposure to THC [112].

Regarding neuroprotective activity, it should be mentioned that CBD induces autophagy. In a neuroblastoma SH-SY5Y cell line and murine astrocytes, the CBD-mediated activation of autophagy was reduced by antagonists of CB1, CB2 and TRPV1 receptors, indicating their involvement in autophagic flux. Autophagy was stimulated through ERK1/2 pathway activation and Akt suppression, revealing new potential cannabinoid-related targets for treating neurodegenerative disorders [210].

Phytocannabinoids also demonstrated neuroprotective effects in experiments mimicking neurodegenerative diseases. In neuronal PC12 cells stimulated with Aβ, CBD reduced ROS levels, lipid peroxidation, intracellular calcium levels, and the induction of the apoptotic cascade [211]. In another study performed on PC12 cells, CBD reduced an Aβ-induced increase in phosphorylated glycogen synthase kinase-3β (pGSK-3β) and tau hyperphosphorylation and also reversed an Aβ-induced decrease in β-catenin expression, suggesting the possible involvement of the Wnt/β-catenin pathway in CBD-mediated neuroprotection [212]. In the same model, CBD inhibited nitrite production and iNOS expression through the inhibition of the p38 pathway and transcription factor NF-ҡB [213]. It seems that the effects of CBD on the reduction in Aβ-induced inflammatory mediators (NO, IL-1β, TNFα, and S100B), the activation of the NF-ҡB pathway, and the death of CA1 pyramidal neurons are mediated by PPARγ receptors [214]. In mice inoculated with human Aβ42, CBD prevented microglial activation, impaired the expression of iNOS and IL-1β, and inhibited NO release [215]. After the intraventricular administration of Aβ in mice, CBD prevented cognitive deficits and the gene expression of IL-6 [216].

Similarly, THC depleted Aβ levels and Aβ aggregation in N2a/AβPP_swe_ cells at very small concentrations, decreased the phosphorylation of pGSK-3β, and enhanced mitochondrial function [217]. In APP/PS1 mice, the intranasal delivery of THC at small doses reduced the production of Aβ and the formation of Aβ oligomers, decreased the activity of GSK-3β and tau phosphorylation, and improved deficits in spatial memory and cognitive decline, although it did not reverse neuropathological hippocampal changes and plasma cytokine levels [218]. Nevertheless, these results indicate that THC could be considered an effective treatment in AD at low doses, particularly in the form of intranasal treatment that minimizes systemic exposure and adverse effects [219].

**Figure 2:**
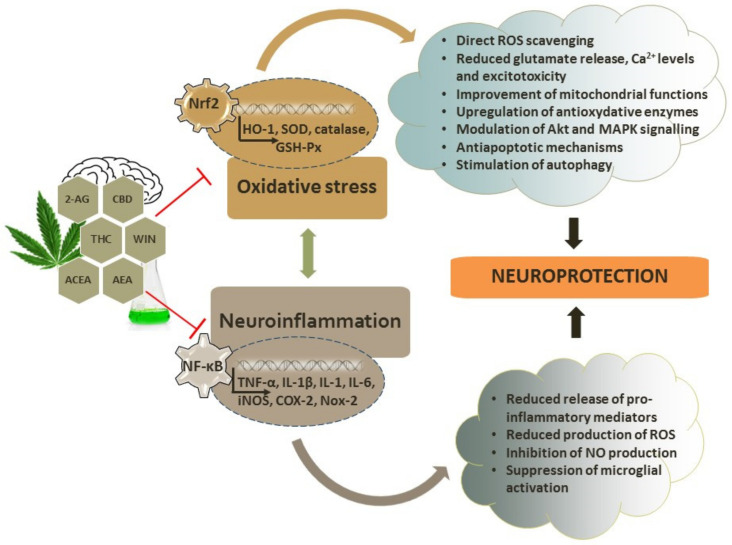
Antioxidative and anti-inflammatory effects of cannabinoids. ACEA—arachidonoyl-2-chloroethylamide; AEA—anandamide; 2-AG—2-arachidonoyl glycerol; CBD—cannabidiol; COX-2—cyclooxygenase 2; HO-1—heme oxygenase-1; GSH-Px—glutathione peroxidase; IL-1β—interleukin (IL)-1β; IL-1—interleukin 1; IL-6—interleukin 6; iNOS—inducible nitric oxide synthase; MAPK—mitogen-activated protein kinase; Nox-2—NADPH oxidase 2; ROS—reactive oxygen species; SOD—superoxide dismutase; TNF-α—tumor necrosis factor alpha; THC—Δ^9^-tetrahydrocannabinol; WIN—WIN 55,212-2.

Similarly, CBD and THC exerted beneficial effects in a 6-hydroxydopamine (6-OHDA)-induced animal model of PD. As both compounds prevented a reduction in dopamine depletion and a decrease in tyrosine hydroxylase activity (and considering that CBD has a very low affinity for CB1 receptors), the beneficial effects were assigned to their direct antioxidative activity [220]. CBD also increased SOD expression in a 6-OHDA-induced animal model of PD [221]. Yet another plant-derived cannabinoid is Δ^9^-tetrahydrocannabivarin (THCV), which activates CB2 receptors and blocks CB1 receptors but also exerts antioxidant properties. Like THC, in 6-OHDA-induced animals, the prolonged administration of THCV reduced the loss of dopaminergic neurons based on its antioxidative activities. Hence, the antioxidative and neuroprotective effects of THCV should be further investigated, particularly when considering that THCV lacks psychoactive side effects. A CBD-enriched plant extract also reduced the loss of dopaminergic neurons in the same study, emphasizing the important contribution of antioxidative mechanisms in neuroprotection (Figure 2) [222].

In rats exposed to 3-nitropropionic acid (a model for HD), CBD reduced 3NP-induced decreases in the levels of SOD1 and SOD2, among other beneficial effects related to GABAergic markers. As two other synthetic cannabinoids, ACEA and HU-308, did not show neuroprotective effects and the CBD effects were independent of the activation of the CB1, TRPV1, and A2A receptors, protection was probably based on the direct antioxidative effects of CBD [223]. Likewise, the THC-mediated activation of CB1 receptors protected mouse striatal neuroblasts against NMDA-induced excitotoxicity by activating the PI3K/Akt/mTORC1/BDNF pathway [83].

**Table 3:** Antioxidant and anti-inflammatory effects of phytocannabinoids in neurodegenerative conditions.

Compound	Signaling	Effects	Model	Ref
CBD	via CB1R	neurogenic effect	mouse model	[43]
THC	-	reduced learning without affecting adult neurogenesis		
THC	greater effects in regions with higher density of CB1R	neuromodulatory effects in brain regions involved in cognitive tasks	human participants	[204]
CBD	↓ LDH release	protection from *tert*-butyl hydroperoxide-induced toxicity	rat cortical neurons	[182]
THC and CBD	-	protection from glutamate toxicity via NMDA, AMPA, and kainate receptors		
CBD	↓ ROS	protection from H_2_O_2_-induced cell death	oligodendrocyte progenitor cells	
CBD	↓ caspase-3 cleavage and apoptosis via mechanisms independent of CB1R, CB2R, TRPV1 or PPAR*γ* receptors	protection from lipopolysaccharide/IFN*γ*-induced cytotoxicity	oligodendrocyte progenitor cells	[208]
	↓ phosphorylation of the eiF2*α* protein	protection from tunicamycin-induced ER stress		
CBD	↓ glutamate level and IL-6, ↓TNF-α, iNOS and COX-2 acting at CB2R and adenosine receptors	protection from hypoxic–ischemic damage	immature mice brain	[209]
THC	reduced number of AMPA receptors following CB1R activation	protection from TNF-α that promotes AMPA receptor-mediated excitotoxicity	rat hippocampal neurons	[112]
CBD	↓ ROS, lipid peroxidation, intracellular calcium and induction of apoptotic cascade	neuroprotection from Aβ-mediated toxicity	PC12 neurons	[211]
CBD	↓ Aβ-induced increase in pGSK-3β and tau hyperphosphorylation and reversed Aβ-induced decrease in β-catenin expression	neuroprotection from Aβ-mediated toxicity	PC12 neurons	[212]
CBD	↓ nitrite production and iNOS through ↓ p38 pathway and NF-ҡB	-	Aβ-stimulated PC12 neurons	[213]
CBD	↓ NO, IL-1β, TNFα, S100B, NF-ҡB pathway via PPARγ receptors	neuroprotection from Aβ and promotion of hippocampal neurogenesis	rat AD model	[214]
CBD	↓ iNOS and IL-1β, NO release	prevention of microglial activation after Aβ inoculation	mouse model	[215]
CBD	↓ IL-6 mRNA in cerebral cortex	improvement of cognitive deficits	Aβ-treated mice	[216]
THC	↓ phosphorylated and total GSK-3β, ↓ Aβ levels, ↑ mitochondrial function	inhibition of Aβ aggregation	N2a/AβPPswe cells	[217]
THC	↓ production of Aβ, ↓ formation of Aβ oligomers, ↓ GSK-3β, ↓ tau phosphorylation	improved spatial memory and cognitive decline	APP/PS1 mice	[218]
CBD and THC	↑ dopamine, ↑ tyrosine hydroxylase mRNA	neuroprotection	6-OHDA-induced mouse model of PD	[220]
CBD	↑ SOD	neuroprotection when immediately administered after the lesion	6-OHDA-induced mice model of PD	[221]
THCV	-	reduced loss of dopaminergic neurons, attenuated motor inhibition	6-OHDA-induced mouse model of PD	[222]
CBD	↓ 3NP-induced decrease in SOD1 and SOD2, restoration of GABA levels	neuroprotection against striatal damage	3-NP-induced rat model of HD	[223]
THC	Activation of PI3K/Akt/mTORC1/BDNF pathway via CB1R	protection from NMDA-induced excitotoxicity	mouse striatal neuroblasts	[83]
THC-rich extracts	↑ glucose utilization and activity of gluconeogenic enzymes; ↑ GSH, SOD and catalase activity, ↓ malondialdehyde and NO levels	improved glucose consumption	isolated rat brain	[224]
THC- and CBD-enriched extract	↓ iNOS, not mediated by CB1 and CB2R	attenuated neurodegeneration and glial activation	malonate-induced rat model of HD	[225]
THC- and CBD-enriched extract	↑ CB2R	small improvements in the progression of neurological deficits	G93A-SOD1 mice model of ALS	[226]
Cannabis	-	moderately effective against appetite loss, depression and spasticity	patients with ALS	[227]

↓, decreased level, expression or activity; ↑, increased level, expression or activity; Aβ, amyloid beta; AD, Alzheimer’s disease; ALS, amyotrophic lateral sclerosis; AMPA, 2-amino-3-(4-butyl-3-hydroxyisoxazol-5-yl)propionic acid; APP/PS, amyloid precursor protein/presenilin; BDNF, brain-derived neurotrophic factor; CB1R, cannabinoid receptor type 1; CBR2, cannabinoid receptor type 2; CBD, cannabidiol; COX-2, cyclooxygenase-2; eiF2α, Eukaryotic Initiation Factor 2; ER, endoplasmic reticulum; GSH, glutathione; GSK-3β, glycogen synthase kinase-3β; HD, Huntington’s disease; IL-1β, interleukin-1β; IL-6, interleukin 6; iNOS, inducible nitric oxide synthase; LDH, lactate dehydrogenase; mTORC1, mammalian target of rapamycin complex 1; NF- ҡB, Nuclear factor kappa-light-chain-enhancer of activated B cells; NMDA, N-methyl-D-aspartate; 3-NP, 3-nitropropionic acid; 6-OHDA, 6-hydroxydopamine; PD, Parkinson’s disease; PPAR, peroxisome proliferator-activated receptor; ROS, reactive oxygen species; SOD, superoxide dismutase; S100B, S100 calcium-binding protein B; THC, Δ^9^-tetrahydrocannabinol; THCV, Δ^9^-tetrahydrocannabivarin; TNF-α, tumor necrosis factor alpha; TRPV1, transient receptor potential vanilloid type 1.

Regarding *Cannabis sativa* extracts, they protected low-density lipoproteins (LDLs) against the Cu^2+^-mediated oxidation. However, the relationship between the biological activity and chemical profile needs further investigation. Samples of female inflorescences from three stable *Cannabis sativa* phenotypes (Strawberry, Exodus Cheese, and Magma) collected at different time points of the flowering period differed in their antioxidant properties, as measured by the ability to reduce the Cu^2+^-induced lipid oxidation of LDL from human plasma. Most of the phytocannabinoids isolated from the extracts functioned as antioxidants, prolonging the latency phase of lipid oxidation. THC was the only cannabinoid able to interfere with the propagation phase, breaking the Cu^2+^-induced lipid oxidation chains in LDL lipids. As previously explained, THC inhibits oxidative stress by scavenging reactive radical species and converting them into more stable, long-living, and less reactive radicals [175]. However, individual cannabinoids were found to be less effective than *Cannabis sativa* extracts, suggesting a synergistic effect of the various phytochemicals present in extracts [174]. In post-ischemic neuronal death, different *Cannabis* extracts, as well as CBD and THC, demonstrated diverse effects, suggesting that an appropriate CBD/THC ratio may be important for efficient therapeutic intervention [2]. Furthermore, THC-rich extracts may promote glucose utilization and the activity of gluconeogenic enzymes in the rat brain [224]. Glucose homeostasis is usually disturbed in neurodegenerative diseases, partially as a result of the oxidative damage of enzymes participating in glycolysis, the Krebs cycle, and oxidative phosphorylation, and the impairment of glucose utilization largely contributes to the progression of pathological changes [159]. THC-rich extracts increased glucose uptake and suppressed oxidative stress levels, as evidenced by enhanced GSH levels, increased SOD and catalase activity, reduced lipid peroxidation, and NO production [224].

In animals administered with malonate, a 1:1 combination of THC- and CBD-enriched botanical extracts attenuated malonate-induced effects on neurodegeneration, glial activation and iNOS expression, but these effects were not mediated by CB1 and CB2 receptors [225]. In a G93A-SOD1 mice model mimicking ALS, a THC- and CBD-enriched plant extract produced only small improvements in the progression of neurological deficits, although it markedly increased CB2 receptors [226].

Cannabis-based products have been introduced into clinical studies for AD and PD, and they have demonstrated small improvements in some secondary symptoms and quality of life. Positive results have mostly been assigned to their anti-inflammatory, immunosuppressive and antioxidant effects, as well as the ability of these products to modulate the ECS [228]. Similarly, in patients with ALS, cannabis was found to be moderately effective against some symptoms (appetite loss, depression, and spasticity) but did not improve speech and swallowing deficits [227]. However, although clinical data on phytocannabinoids and neurodegenerative diseases are not too encouraging regarding primary symptoms, the antioxidative potential of plant-derived cannabinoids in the improvement of redox balance and perhaps the alleviation of the symptoms of the disease should be further investigated, particularly if they are applied early during the disease and in optimized combinations of bioactive phytocannabinoids.

### 4.3. Antioxidative and Neuroprotective Effects of Synthetic Cannabinoids

Synthetic cannabinoids have also demonstrated neuroprotective effects (Table 4). WIN 55,212-2 is the nonselective cannabinoid receptor agonist acting on both CB1 and CB2 receptors, and it is very often used as an analogue of endocannabinoids. It is particularly efficient in inhibiting voltage-dependent calcium channels [45] and presynaptic glutamate release [36], offering protection against excitotoxicity and cell death. In hippocampal neurons, WIN 55,212-2 was shown to attenuate NMDA-induced increases in intracellular calcium levels and to reduce neuronal death by inhibiting adenylyl cyclase and reducing intracellular calcium levels via the cAMP/PKA pathway. Both effects were prevented by the selective CB1 receptor antagonist SR141716A [113]. In the TNF-α-induced excitotoxicity that is mediated by an increased number of surface AMPA receptors, WIN 55,212-2 exerted neuroprotective effects by interfering with receptor trafficking after CB1 receptor activation [112]. Neuronal death and ROS formation in cortical neuron cultures exposed to FeCl_2_ were also reduced by WIN 55,212-2. Similarly, protection was achieved through the activation of CB1 receptors and PKA inhibition [229]. In cultured cortical neurons in vitro and mice brains in vivo, WIN 55,212 attenuated NMDA-induced neuronal death. NOS inhibitors, 7-nitroindazole (7-NI), and N-nitro-L-arginine methyl ester (L-NAME) also attenuated the cytotoxic effect of NMDA, and dibutyryl-cAMP (a PKA activator) blocked the protective effect of R(+)-WIN 55,212, thus indicating that its neuroprotective effects were, at least in part, achieved through the activation of CB1 receptors, the downstream inhibition of PKA, and a reduction in NO production [230].

In the brain synaptosomes of adult and adolescent rats exposed to several organic acids (glutaric acid, 3-hydroxyglutaric acid, methylmalonic acid and propionic acid) as a model of organic acidemias (hereditary metabolic disorders accompanied by oxidative stress, excitotoxicity and neurodegeneration), pre-treatment with the WIN 55,212-2 improved mitochondrial impairment and prevented the production of ROS and lipid peroxidation without exerting noxious effects [231]. As the ECS is involved in the regulation of NMDA receptors, the authors concluded that the WIN 55,212-2 effects were probably mediated by the modulation of the postsynaptic NMDA receptors, although they could not exclude the possibility of the direct antioxidant activities of WIN 55,212-2.

In primary dorsal root ganglia neurons and neuronal F-11 cells, pre-treatment with WIN 55,212-2 attenuated NMDA-induced calcium influx and cell death through the inositol triphosphate (IP_3_) signaling pathway. Both effects were prevented by SR141716A but not the CB2 receptor antagonist SR144528 [232]. On the other hand, the acute application of WIN 55,212-2 alone and the activation of the CB1 receptor evoked a dose-dependent rise of intracellular calcium. To distinguish whether WIN 55,212-2- and NMDA-induced calcium increases originate from the intracellular or extracellular calcium stores, specific inhibitors were used. The WIN 55,212-2-induced increase was reduced with the thapsigargin, the ER Ca^2+^ pump inhibitor, suggesting that the Ca^2+^ released from the ER contributed to the WIN 55,212-2-elicited calcium increase. On the contrary, the NMDA-induced increase in intracellular calcium levels was not affected by thapsigargin but was reduced by the removal of extracellular calcium. More importantly, the WIN 55,212-2-induced increase was required to inhibit the NMDA-mediated Ca^2+^ influx and prevent the cytotoxic effect of NMDA [232]. As 2-aminoethyl diphenylborinate, an inhibitor of the IP_3_ signaling pathway, also prevented the effect of WIN 55,212-2 on NMDA-elicited Ca^2+^ influx, it was concluded that the WIN 55,212-2-initiated IP_3_ signaling and calcium release from the intracellular stores are critical for WIN 55,212-2-mediated neuroprotection [232]. Therefore, Ca^2+^, as a signaling molecule, may have opposite effects, probably depending on the intensity of the calcium increase, the temporal pattern of the increase, and cannabinoid ligands [232]. Other studies have shown that the inactivation of NMDA receptors in response to high levels of cytoplasmic calcium is mediated by Ca^2+^-dependent signaling proteins such as calcineurin and calmodulin [233]. However, in cerebellar granule neurons, methanandamide, WIN, and HU-210 (the non-selective cannabinoid agonist) were shown to enhance the peak amplitude of the Ca^2+^ response elicited by the stimulation of the NMDA receptors. As the effect was blocked by the Gi/Go protein inhibitor PTX and inhibited by SR141716A, it was likely mediated by the cannabinoid receptors. Further studies revealed that cannabinoids release Ca^2+^ from IP_3_-sensitive intracellular stores, suggesting the important modulatory role of cannabinoids in the regulation of intracellular Ca^2+^ signaling [119].

Regarding AD, in the presence of WIN 55,212-2, anandamide hydrolysis was shown to be reduced, enhancing anandamide levels [132]. WIN 55,212-2 also prevented microglial activation and improved cognitive deficits in rats administered with Aβ, whereas WIN 55,212-2, HU-210 and JWH-133 (the CB2 selective agonist) counteracted Aβ-induced microglial activation and the enhancement of TNF-α release in a pure microglial cell culture, suggesting that the neuroprotective effects of cannabinoids against Aβ-mediated toxicity greatly relies on the inhibition of microglial activation [105]. In yet another study in transgenic APP mice, the beneficial effects of the cannabinoids WIN 55,212-2 and JWH-133 were assigned to anti-inflammatory effects (reduction in COX-2 levels and TNF-α mRNA expression) and an increase in Aβ clearance [234]. Similarly, HU-210 protected mouse cerebellar granule cells from 6-OHDA-mediated toxicity by modulating glial function [220]. In a similar study, the effects of the synthetic cannabinoids ACEA, HU-308 (the CB2 receptor agonist) and WIN 55,212-2 were investigated in rats with 6-OHDA-induced unilateral lesions of nigrostriatal dopaminergic neurons. Only HU-308 exerted a small effect, indicating a more prominent contribution of CB2 receptors in neuroprotection [221]. The only compound that exerted a significant recovery was AM404, which is known to inhibit the inactivation of endocannabinoids by blocking endocannabinoid transport. However, the protective effect of AM404 was ultimately assigned to its direct antioxidative properties, as CBD also attenuated 6-OHDA-induced dopamine loss. The neuroprotective effect was accompanied by the overexpression of SOD mRNA. These results suggest that endocannabinoids able to exert antioxidative effects independently from the CB1 receptor activation hold great potential as neuroprotective agents against 6-OHDA-induced damage [221]. In another study, the effect of WIN 55,212-2 on L-DOPA-induced motor disabilities in 6-OHDA-lesioned rats was studied. Motor deficits were reduced via the chronic administration of WIN 55,212-2, as well as L-DOPA-mediated alterations in ERK1/2 activation [235]. Furthermore, in an MPTP-induced model of PD, the activation of CB1 receptors with WIN 55,212-2 or HU210 improved the survival of dopaminergic neurons by suppressing the activation of microglial NADPH oxidase, the release of the pro-inflammatory cytokines IL-1β and TNF-α, ROS production, and the oxidative damage of proteins and nucleic acids [236]. However, in a similar study, WIN 55,212-2 was also effective against MPTP-induced toxicity in mice; it recovered dopamine levels and attenuated microglial activation, but the protection of dopaminergic neurons was independent from the activation of CB1 receptors and the beneficial effects of WIN 55,212-2 were assigned to the activation of CB2 receptors [237]. HU210 also protected mouse striatal cells against NMDA-induced excitotoxicity by activating the PI3K/Akt/mTORC1/BDNF pathway [83].

In addition, the WIN 55,212-2-mediated activation of CB1 receptors protected astrocytes, both in vitro and in vivo, from ceramide-induced apoptosis. The anti-apoptotic effect was mediated by CB1 receptors and the PI3K/Akt and ERK-signaling pathways, whereby the activation of ERK and its downstream target p90 ribosomal S6 kinase was probably PI3K-induced [86].

In a G93A-SOD1 mice model of ALS, WIN 55,212-2 delayed the progression of the disease. In the same model, the genetic ablation of FAAH also prevented the appearance of the clinical signs of the disease, whereas the ablation of CB1 receptors did not have any effect, suggesting a more prominent contribution of CB2 receptors in neuroprotection, at least in this model [196]. Similar effects were observed for the CB2 agonist AM-1241, indicating an important contribution of CB2 receptor in reducing inflammation and disease onset [238]. HU-308, the synthetic selective CB2 receptor agonist, was shown to reduce the levels of TNF-α and gliosis in malonate-induced toxicity, further suggesting the important contribution of the activation of CB2 receptors in neuroprotection [54].

Likewise, the CB2 receptor agonist JWH133 was found to exert neuroprotective effects in SH-SY5Y cells exposed to MPP^+^ [194]. JWH133 also protected hippocampal neurons against Aβ-induced injury. Pre-treatment with JWH133 prevented the suppression of Akt signaling, reversed the Bcl-2/Bax ratio, and counteracted increases in caspase-3 activity [239].

The effects of ACEA were investigated in Neuro-2a cells exposed to LPS or tunicamycin, a potent inducer of ER stress. ACEA protected Neuro-2a cells from both neuroinflammation and ER stress, although the protective effect against tunicamycin was not mediated by the CB1 receptor but was achieved through the TRPV1 receptor and the ERK1/2 signaling pathway [19]. In a mouse model of cerebral ischemia/reperfusion injury, ACEA upregulated the expression of mitochondrial CB1 receptors in the hippocampus, whereas in cultured hippocampal neurons exposed to oxygen-glucose deprivation/reoxygenation, ACEA decreased the production of ROS and the apoptotic rate and improved mitochondrial function, at least partially by acting at the mitochondrial CB1 receptors [100].

Hu-211, yet another synthetic cannabinoid derivative, also demonstrated neuroprotective effects, though it was found to act as a non-competitive antagonist of NMDA receptors and a free radical scavenger. Perhaps the further development of such compounds (with the two beneficial activities in one molecule) could be a promising approach when considering (synthetic) cannabinoids as therapeutic agents against neurodegeneration [240].

**Table 4:** Antioxidant and anti-inflammatory effects of synthetic cannabinoids in neurodegenerative conditions.

Compound	Signaling	Effects	Model	Ref.
WIN 55,212-2	↓ adenylyl cyclase and ↓ intracellular calcium via CB1R and cAMP/PKA pathway	protection from NMDA-induced neuronal death	hippocampal neurons	[113]
WIN 55,212-2	interfering with AMPA receptor trafficking after CB1R activation	protection from TNF-α-induced excitotoxicity	hippocampal neurons	[112]
WIN 55,212-2	↓ ROS formation through CB1R and PKA inhibition	protection from FeCl_2_-induced neuronal death	cortical neuron cultures	[229]
WIN 55,212	activation of CB1R, ↓ PKA activity and NO production	protection from NMDA-induced cell death	cultured cortical neurons and mice brains	[230]
WIN 55,212-2	↑ mitochondrial function, ↓ ROS and lipid peroxidation	protection from organic acidemias	rat brain synaptosomes	[231]
WIN 55,212-2	↓ NMDA-induced calcium influx via IP_3_ signaling	protection from NMDA-induced cell death	primary dorsal root ganglia neurons and F-11 neurons	[232]
WIN 55,212-2	-	prevention of cognitive impairment and microglial activation	rats administered with Aβ	[105]
WIN 55,212-2, HU-210, JWH-133	↓ TNF-α release	↓ microglial activation	microglial cell culture	
WIN 55,212-2 and JWH-133	↓ COX-2, TNF-α mRNA, ↑ Aβ clearance	JWH-133 normalized novel object recognition and ↓ microglial activation	transgenic APP mice	[234]
HU-210	-	modulation of glial function, protection from 6-OHDA-mediated toxicity	mouse cerebellar granule cells	[220]
ACEA	-	no neuroprotection	6-OHDA-induced lesions in mice	[221]
HU-308	-	modest neuroprotection		
WIN 55,212-2	-	no neuroprotection		
WIN 55,212-2	modulation of L-DOPA-induced ERK1/2 activation	attenuation of L-DOPA-induced motor disabilities	6-OHDA-induced rat model of PD	[235]
WIN 55,212-2 and HU210	↓ NADPH oxidase activation, IL-1β and TNF-α release, ROS production and oxidative damage of proteins and nucleic acids	improved survival of dopaminergic neurons	MPTP-induced model of PD	[236]
HU210	↑ PI3K/Akt/mTORC1/BDNF	protection from NMDA-induced excitotoxicity	mouse striatal neurons	[83]
WIN 55,212-2	↑ dopamine levels via CB2R	↓ microglial activation, protection against loss of dopaminergic neurons	MPTP-induced toxicity in mice	[237]
ACEA	via CB1R (for LPS) or via TRPV1 channel (for tunicamycin) modulation of ERK1/2 pathway	protection from neuroinflammation and endoplasmic reticulum stress	Neuro-2a cells exposed to LPS or tunicamycin	[19]
ACEA	↓ ROS and apoptotic rate	improved mitochondrial function	hippocampal neurons exposed to oxygen-glucose deprivation/reoxygenation	[100]
WIN 55,212-2	↓ apoptosis via CB1R, PI3K/Akt and ERK pathways	protection from ceramide-induced apoptosis in vitro and in vivo	astrocytes	[86]
WIN 55,212-2	-	no appearance of the clinical signs of the disease	G93A-SOD1 mice model of ALS	[196]
AM-1241	-	↑ survival interval after disease onset	G93A-SOD1 mice model of ALS	[238]
HU-308	↓ TNF-α and gliosis via CB2R	neuroprotection from malonate-induced toxicity	malonate-induced rat model of HD	[54]
JWH133	via CB2R	neuroprotection from MPP^+^-induced toxicity	SH-SY5Y cells	[194]
JWH133	prevented suppression of Akt signaling, reversed Bcl-2/Bax ratio and prevented caspase-3 increase	protection from Aβ-induced injury	hippocampal neurons	[239]
NP137	↓ BACE1 activity	neuroprotection from Aβ	Aβ-treated primary cortical neurons	[241]
	-	improvement of spatial navigation	TgAPP mice	
NITyr	via CB1R, ↓ ROS generation, and ↑ autophagy-related proteins and autophagy	attenuated H_2_O_2_-induced neurotoxic effects	rat pheochromocytoma PC12 cells	[185]
NITyr	↑ BDNF and autophagy by CB2/AMPK/mTOR/ULK1	attenuated Aβ-induced toxicity	primary cortical neurons	[186]

↓, decreased level, expression or activity; ↑, increased level, expression or activity; Aβ, amyloid beta; ACEA, Arachidonyl-2′-chloroethylamide; AD, Alzheimer’s disease; ALS, amyotrophic lateral sclerosis; AMPA, 2-amino-3-(4-butyl-3-hydroxyisoxazol-5-yl)propionic acid; AMPK, 5′ AMP-activated protein kinase; APP, amyloid precursor protein; BACE1, β-secretase; BDNF, brain-derived neurotrophic factor; CB1R, cannabinoid receptor type 1; CBR2, cannabinoid receptor type 2; COX-2, cyclooxygenase-2; HD, Huntington’s disease; IL-1β, interleukin-1β; IP3, inositol trisphosphate; iNOS, inducible nitric oxide synthase; L-DOPA, l-3,4-dihydroxyphenylalanine; LPS, lipopolysaccharide; NMDA, N-methyl-D-aspartate; MPP^+^, 1-methyl-4-phenylpyridinium; MPTP, 1-methyl-4-phenyl-1,2,3,6-tetrahydropyridine; mTOR, mammalian target of rapamycin; 3-NP, 3-nitropropionic acid; 6-OHDA, 6-hydroxydopamine; PD, Parkinson’s disease; PKA, protein kinase A; ROS, reactive oxygen species; TNF-α, tumor necrosis factor alpha; TRPV1, transient receptor potential vanilloid type 1, ULK1, Unc-51 Like Autophagy Activating Kinase 1.

Recently, a CB1/CB2 receptor agonist, an indazolylketone derivative with a multitarget profile, was designed. In a cellular AD model, this compound inhibited the activity of BACE1, a transmembrane peptidase critically implicated in Aβ production. The compound termed NP137 also showed neuroprotective effects in Aβ-treated primary cortical neurons and improved spatial navigation in TgAPP mice, indicating that multitarget cannabinoids could be a promising therapeutic strategy for the treatment of AD and possibly other neurodegenerative conditions [241]. In addition, the NP137-mediated activation of CB1 receptors normalized the response of lymphoblasts from AD patients to serum stimulation and withdrawal. In the presence of serum, NP137 prevented the overactivation of PI3K/Akt signaling in AD cells, whereas under serum deprivation, NP137 normalized phosphorylation and the levels of ERK1/2, as well as the expression and subcellular localization of p21, sensitizing AD cells to apoptosis [241]. This further means that the activation of CB receptors functionally regulates the activation of different signaling pathways depending on the cellular and environmental context, indicating the ECS to be a reliable, although complex, target in neuroprotection.

Long noncoding RNA (lncRNA), together with microRNAs, are linked to the development of AD. One of these lncRNAs termed metastasis-associated lung adenocarcinoma transcript 1 (MALAT1) was markedly downregulated in AD rats and PC12 and C6 cells following Aβ25-35 treatment. miR-30b was identified as a target miRNA of MALAT1 and was aberrantly overexpressed in AD rats and PC12 and C6 cells. miR-30b directly binds to *CNR1* mRNA and downregulates its expression. Accordingly, the overexpression of MALAT1 or *CNR1* was found to exert neuroprotective effects. It improved the survival of PC12 and C6 cells, reduced the neuronal injury of hippocampal tissue and improved performance in the Morris water maze, reduced the expression of the pro-inflammatory cytokines IL-6 and TNF-α, increased the expression of the anti-inflammatory cytokine IL-10, and enhanced the activity of the PI3K/Akt signaling pathway [242], thus offering new possibilities in cannabinoid-related AD pharmacotherapy at the gene level.

## 5. Conclusions

Cannabinoids comprise a structurally diverse family of compounds that act on various molecular targets, modulating a wide range of biological activities. Due to their lipophilic nature, cannabinoids can easily pass the blood–brain barrier and exert their effects in the brain. In various neurodegenerative conditions, the production of endocannabinoids and the expressional pattern of cannabinoid receptors have been disturbed, probably contributing to pathophysiological changes. Hence, the ECS has been appreciated as a possible target in neuroprotection at various levels. A growing amount of evidence suggests that different ligands targeting cannabinoid receptors could affect distinct signaling pathways and exert different biological responses, which needs to be clarified in future studies when considering cannabinoids as potential therapeutics.

The neuroprotective effects of cannabinoids are mediated by the activation of CB1 receptors, the regulation of glutamate release and cytosolic calcium levels, and excitotoxicity, as well as direct and indirect antioxidative effects, anti-inflammatory properties, and the stimulation of neurogenesis, all of which may contribute to the more efficient outcomes of pharmacological interventions. CBD, a plant-derived cannabinoid, is particularly interesting because it is devoid of psychoactive effects, could be applied at higher doses, and possesses intrinsic antioxidative properties. The great anti-inflammatory potential of CB2 signaling due to the suppression of ROS production and pro-inflammatory mediators makes it another reasonable approach in neuroprotection. Targeting CB2 receptors and downstream signaling pathways by selective CB2 agonists may reduce oxidative/nitrosative stress by attenuating microglial activation and neuroinflammation.

Despite the many performed studies, the effects of cannabinoids in oxidative stress conditions are far from conclusive. Further studies are needed to assess the full potential of cannabinoids in neurodegenerative conditions accompanied by the overproduction of ROS and the overactivation of distinct redox-sensitive signaling pathways. With a better understanding of the pathways involved in the neuroprotective effects of cannabinoids, it would be possible to design more effective cannabinoid-based therapies. CB2 receptors seem to be a more appropriate target because they offer the possibility of non-psychotropic interventions. Cannabinoid-based therapies without psychoactive properties could be a promising therapeutic approach in neurodegenerative diseases that is worth further study.

## Data Availability

Not applicable.

## References

[1] Friedman D., Sirven J.I. (2017). Historical perspective on the medical use of cannabis for epilepsy: Ancient times to the 1980s. Epilepsy Behav..

[2] Landucci E., Mazzantini C., Lana D., Davolio P.L., Giovannini M.G., Pellegrini-Giampietro D.E. (2021). Neuroprotective Effects of Cannabidiol but Not Δ^9^-Tetrahydrocannabinol in Rat Hippocampal Slices Exposed to Oxygen-Glucose Deprivation: Studies with *Cannabis* Extracts and Selected Cannabinoids. Int. J. Mol. Sci..

[3] Giuliano C., Francavilla M., Ongari G., Petese A., Ghezzi C., Rossini N., Blandini F., Cerri S. (2021). Neuroprotective and Symptomatic Effects of Cannabidiol in an Animal Model of Parkinson’s Disease. Int. J. Mol. Sci..

[4] Maroon J., Bost J. (2018). Review of the neurological benefits of phytocannabinoids. Surg. Neurol. Int..

[5] Brigida A.L., Schultz S., Cascone M., Antonucci N., Siniscalco D. (2017). Endocannabinoid Signal Dysregulation in Autism Spectrum Disorders: A Correlation Link between Inflammatory State and Neuro-Immune Alterations. Int. J. Mol. Sci..

[6] Morris G., Walder K., Kloiber S., Amminger P., Berk M., Bortolasci C.C., Maes M., Puri B.K., Carvalho A.F. (2021). The endocannabinoidome in neuropsychiatry: Opportunities and potential risks. Pharmacol. Res..

[7] Zou S., Kumar U. (2018). Cannabinoid Receptors and the Endocannabinoid System: Signaling and Function in the Central Nervous System. Int. J. Mol. Sci..

[8] Paloczi J., Varga Z.V., Hasko G., Pacher P. (2018). Neuroprotection in Oxidative Stress-Related Neurodegenerative Diseases: Role of Endocannabinoid System Modulation. Antioxid. Redox Signal..

[9] Haspula D., Clark M.A. (2020). Cannabinoid Receptors: An Update on Cell Signaling, Pathophysiological Roles and Therapeutic Opportunities in Neurological, Cardiovascular, and Inflammatory Diseases. Int. J. Mol. Sci..

[10] Pertwee R.G., Howlett A.C., Abood M.E., Alexander S.P.H., Di Marzo V., Elphick M.R., Greasley P.J., Hansen H.S., Kunos G., Mackie K. (2010). International Union of Basic and Clinical Pharmacology. LXXIX. Cannabinoid receptors and their ligands: Beyond CB1 and CB2. Pharmacol. Rev..

[11] Sánchez-Blázquez P., Rodríguez-Muñoz M., Garzón J. (2013). The cannabinoid receptor 1 associates with NMDA receptors to produce glutamatergic hypofunction: Implications in psychosis and schizophrenia. Front. Pharmacol..

[12] Rios C., Gomes I., Devi L.A. (2006). μ opioid and CB1 cannabinoid receptor interactions: Reciprocal inhibition of receptor signaling and neuritogenesis. Br. J. Pharmacol..

[13] Sun Y., Alexander S.P., Garle M.J., Gibson C.L., Hewitt K., Murphy S.P., Kendall D.A., Bennett A.J. (2007). Cannabinoid activation of PPAR alpha; a novel neuroprotective mechanism. Br. J. Pharmacol..

[14] Palazzos E., de Novellis V., Marabese I., Rossi F., Maione S. (2006). Metabotropic glutamate and cannabinoid receptor crosstalk in periaqueductal grey pain processing. Curr. Neuropharmacol..

[15] Lauckner J.E., Jensen J.B., Chen H.Y., Lu H.C., Hille B., Mackie K. (2008). GPR55 is a cannabinoid receptor that increases intracellular calcium and inhibits M current. Proc. Natl. Acad. Sci. USA.

[16] Sylantyev S., Jensen T.P., Ross R.A., Rusakov D.A. (2013). Cannabinoid- and lysophosphatidylinositol-sensitive receptor GPR55 boosts neurotransmitter release at central synapses. Proc. Natl. Acad. Sci. USA.

[17] Soderstrom K., Soliman E., Van Dross R. (2017). Cannabinoids Modulate Neuronal Activity and Cancer by CB1 and CB2 Receptor-Independent Mechanisms. Front. Pharmacol..

[18] Muller C., Morales P., Reggio P.H. (2019). Cannabinoid ligands targeting TRP channels. Front. Mol. Neurosci..

[19] Vrechi T.A., Crunfli F., Costa A.P., Torrão A.S. (2018). Cannabinoid Receptor Type 1 Agonist ACEA Protects Neurons from Death and Attenuates Endoplasmic Reticulum Stress-Related Apoptotic Pathway Signaling. Neurotox. Res..

[20] Im D.-S. (2021). GPR119 and GPR55 as Receptors for Fatty Acid Ethanolamides, Oleoylethanolamide and Palmitoylethanolamide. Int. J. Mol. Sci..

[21] Felder C.C., Briley E.M., Axelrod J., Simpson J.T., Mackie K., Devane W.A. (1993). Anandamide, an endogenous cannabimimetic eicosanoid, binds to the cloned human cannabinoid receptor and stimulates receptor-mediated signal transduction. Proc. Natl. Acad. Sci. USA.

[22] Gonsiorek W., Lunn C., Fan X., Narula S., Lundell D., Hipkin R.W. (2000). Endocannabinoid 2-arachidonyl glycerol is a full agonist through human type 2 cannabinoid receptor: Antagonism by anandamide. Mol. Pharmacol..

[23] Hampson A.J., Hill W.A., Zan-Phillips M., Makriyannis A., Leung E., Eglen R.M., Bornheim L.M. (1995). Anandamide hydroxylation by brain lipoxygenase: Metabolite structures and potencies at the cannabinoid receptor. Biochim. Biophys. Acta.

[24] Turcotte C., Chouinard F., Lefebvre J.S., Flamand N. (2015). Regulation of inflammation by cannabinoids, the endocannabinoids 2-arachidonoyl-glycerol and arachidonoyl-ethanolamide, and their metabolites. J. Leukoc. Biol..

[25] Cristino L., Bisogno T., Di Marzo V. (2020). Cannabinoids and the expanded endocannabinoid system in neurological disorders. Nat. Rev. Neurol..

[26] Sridar C., Snider N.T., Hollenberg P.F. (2011). Anandamide oxidation by wild-type and polymorphically expressed CYP2B6 and CYP2D6. Drug Metab. Dispos..

[27] Snider N.T., Nast J.A., Tesmer L.A., Hollenberg P.F. (2009). A cytochrome P450-derived epoxygenated metabolite of anandamide is a potent cannabinoid receptor 2-selective agonist. Mol. Pharmacol..

[28] Cravatt B.F., Demarest K., Patricelli M.P., Bracey M.H., Giang D.K., Martin B.R., Lichtman A.H. (2001). Supersensitivity to anandamide and enhanced endogenous cannabinoid signaling in mice lacking fatty acid amide hydrolase. Proc. Natl. Acad. Sci. USA.

[29] Mecha M., Feliú A., Carrillo-Salinas F.J., Rueda-Zubiaurre A., Ortega-Gutiérrez S., de Sola R.G., Guaza C. (2015). Endocannabinoids drive the acquisition of an alternative phenotype in microglia. Brain Behav. Immun..

[30] Clement A.B., Hawkins E.G., Lichtman A.H., Cravatt B.F. (2003). Increased seizure susceptibility and proconvulsant activity of anandamide in mice lacking fatty acid amide hydrolase. J. Neurosci..

[31] Valdeolivas S., Pazos M.R., Bisogno T., Piscitelli F., Iannotti F.A., Allarà M., Sagredo O., Di Marzo V., Fernández-Ruiz J. (2013). The inhibition of 2-arachidonoyl-glycerol (2-AG) biosynthesis, rather than enhancing striatal damage, protects striatal neurons from malonate-induced death: A potential role of cyclooxygenase-2-dependent metabolism of 2-AG. Cell Death Dis..

[32] Gabrielli M., Battista N., Riganti L., Prada I., Antonucci F., Cantone L., Lombardi M., Matteoli M., Maccarrone M., Verderio C. (2015). Active endocannabinoids are secreted on the surface of microglial microvesicles. SpringerPlus.

[33] Gabrielli M., Battista N., Riganti L., Prada I., Antonucci F., Cantone L., Matteoli M., Maccarrone M., Verderio C. (2015). Active endocannabinoids are secreted on extracellular membrane vesicles. EMBO Rep..

[34] Oddi S., Fezza F., Pasquariello N., De Simone C., Rapino C., Dainese E., Finazzi-Agrò A., Maccarrone M. (2008). Evidence for the intracellular accumulation of anandamide in adiposomes. Cell. Mol. Life Sci..

[35] Maccarrone M. (2017). Metabolism of the Endocannabinoid Anandamide: Open Questions after 25 Years. Front. Mol. Neurosci..

[36] Shen M., Piser T.M., Seybold V.S., Thayer S.A. (1996). Cannabinoid receptor agonists inhibit glutamatergic synaptic transmission in rat hippocampal cultures. J. Neurosci..

[37] Szabo B., Schlicker E. (2005). Effects of cannabinoids on neurotransmission. Handb. Exp. Pharmacol..

[38] Sánchez-Blázquez P., Rodríguez-Muñoz M., Vicente-Sánchez A., Garzón J. (2013). Cannabinoid receptors couple to NMDA receptors to reduce the production of NO and the mobilization of zinc induced by glutamate. Antioxid. Redox Signal..

[39] Herkenham M., Lynn A.B., Johnson M.R., Melvin L.S., de Costa B.R., Rice K.C. (1991). Characterization and localization of cannabinoid receptors in rat brain: A quantitative in vitro autoradiographic study. J. Neurosci..

[40] Carriba P., Ortiz O., Patkar K., Justinova Z., Stroik J., Themann A., Müller C., Woods A.S., Hope B.T., Ciruela F. (2007). Striatal adenosine A2A and cannabinoid CB1 receptors form functional heteromeric complexes that mediate the motor effects of cannabinoids. Neuropsychopharmacology.

[41] Carlson G., Wang Y., Alger B.E. (2002). Endocannabinoids facilitate the induction of LTP in the hippocampus. Nat. Neurosci..

[42] Jin K., Xie L., Kim S.H., Parmentier-Batteur S., Sun Y., Mao X.O., Childs J., Greenberg D.A. (2004). Defective adult neurogenesis in CB1 cannabinoid receptor knockout mice. Mol. Pharmacol..

[43] Wolf S.A., Bick-Sander A., Fabel K., Leal-Galicia P., Tauber S., Ramirez-Rodriguez G., Müller A., Melnik A., Waltinger T.P., Ullrich O. (2010). Cannabinoid receptor CB1 mediates baseline and activity-induced survival of new neurons in adult hippocampal neurogenesis. Cell Commun. Signal..

[44] Rueda D., Navarro B., Martinez-Serrano A., Guzman M., Galve-Roperh I. (2002). The endocannabinoid anandamide inhibits neuronal progenitor cell differentiation through attenuation of the Rap1/B-Raf/ERK pathway. J. Biol. Chem..

[45] Twitchell W., Brown S., Mackie K. (1997). Cannabinoids inhibit N- and P/Q-type calcium channels in cultured rat hippocampal neurons. J. Neurophysiol..

[46] Liu J., Gao B., Mirshahi F., Sanyal A.J., Khanolkar A.D., Makriyannis A., Kunos G. (2000). Functional CB1 cannabinoid receptors in human vascular endothelial cells. Biochem. J..

[47] Molina-Holgado E., Vela J.M., Arévalo-Martín A., Almazán G., Molina-Holgado F., Borrell J., Guaza C. (2002). Cannabinoids promote oligodendrocyte progenitor survival: Involvement of cannabinoid receptors and phosphatidylinositol-3 kinase/Akt signaling. J. Neurosci..

[48] Freundt-Revilla J., Kegler K., Baumgärtner W., Tipold A. (2017). Spatial distribution of cannabinoid receptor type 1 (CB1) in normal canine central and peripheral nervous system. PLoS ONE.

[49] Croci T., Manara L., Aureggi G., Guagnini F., Rinaldi-Carmona M., Maffrand J.-P., Fur G., Mukenge S., Ferla G. (1998). In Vitro functional evidence of neuronal cannabinoid CB _1_ receptors in human ileum. Br. J. Pharmacol..

[50] Kulkarni-Narla A., Brown D.R. (2000). Localization of CB1-cannabinoid receptor immunoreactivity in the porcine enteric nervous system. Cell Tissue Res..

[51] Camilleri M. (2018). Cannabinoids and gastrointestinal motility: Pharmacology, clinical effects, and potential therapeutics in humans. Neurogastroenterol. Motil..

[52] Maresz K., Carrier E.J., Ponomarev E.D., Hillard C.J., Dittel B.N. (2005). Modulation of the cannabinoid CB2 receptor in microglial cells in response to inflammatory stimuli. J. Neurochem..

[53] Komorowska-Müller J.A., Schmöle A.-C. (2021). CB2 Receptor in Microglia: The Guardian of Self-Control. Int. J. Mol. Sci..

[54] Sagredo O., González S., Aroyo I., Pazos M.R., Benito C., Lastres-Becker I., Romero J.P., Tolón R.M., Mechoulam R., Brouillet E. (2009). Cannabinoid CB2 receptor agonists protect the striatum against malonate toxicity: Relevance for Huntington’s disease. Glia.

[55] Rivas-Santisteban R., Lillo A., Lillo J., Rebassa J.B., Contestí J.S., Saura C.A., Franco R., Navarro G. (2021). N-Methyl-D-aspartate (NMDA) and cannabinoid CB_2_ receptors form functional complexes in cells of the central nervous system: Insights into the therapeutic potential of neuronal and microglial NMDA receptors. Alzheimers Res. Ther..

[56] Rhee M.H., Bayewitch M., Avidor-Reiss T., Levy R., Vogel Z. (1998). Cannabinoid receptor activation differentially regulates the various adenylyl cyclase isozymes. J. Neurochem..

[57] Zhou J., Noori H., Burkovskiy I., Lafreniere J.D., Kelly M.E.M., Lehmann C. (2019). Modulation of the Endocannabinoid System Following Central Nervous System Injury. Int. J. Mol. Sci..

[58] Kibret B.G., Ishiguro H., Horiuchi Y., Onaivi E.S. (2022). New Insights and Potential Therapeutic Targeting of CB2 Cannabinoid Receptors in CNS Disorders. Int. J. Mol. Sci..

[59] Cassano T., Calcagnini S., Pace L., De Marco F., Romano A., Gaetani S. (2017). Cannabinoid Receptor 2 Signaling in Neurodegenerative Disorders: From Pathogenesis to a Promising Therapeutic Target. Front. Neurosci..

[60] Van Sickle M.D., Duncan M., Kingsley P.J., Mouihate A., Urbani P., Mackie K., Stella N., Makriyannis A., Piomelli D., Davison J.S. (2005). Identification and functional characterization of brainstem cannabinoid CB2 receptors. Science.

[61] Zhang J., Hoffert C., Vu H.K., Groblewski T., Ahmad S., O’Donnell D. (2003). Induction of CB2 receptor expression in the rat spinal cord of neuropathic but not inflammatory chronic pain models. Eur. J. Neurosci..

[62] Yuste J.E., Tarragon E., Campuzano C.M., Ros-Bernal F. (2015). Implications of glial nitric oxide in neurodegenerative diseases. Front. Cell. Neurosci..

[63] Ehrhart J., Obregon D., Mori T., Hou H., Sun N., Bai Y., Klein T., Fernandez F., Tan J., Shytle R.D. (2005). Stimulation of cannabinoid receptor 2 (CB2) suppresses microglial activation. J. Neuroinflammation.

[64] Ma L., Jia J., Liu X., Bai F., Wang Q., Xiong L. (2015). Activation of murine microglial N9 cells is attenuated through cannabinoid receptor CB2 signaling. Biochem. Biophys. Res. Commun..

[65] Nogueras-Ortiz C., Yudowski G.A. (2016). The multiple waves of cannabinoid 1 receptor signaling. Mol. Pharmacol..

[66] Howlett A., Qualy J.M., Khachatrian L.L. (1986). Involvement of Gi in the inhibition of adenylate cyclase by cannabimimetic drugs. Mol. Pharmacol..

[67] Childers S.R., Deadwyler S.A. (1996). Role of cyclic AMP in the actions of cannabinoid receptors. Biochem. Pharmacol..

[68] Hampson A.J., Grimaldi M. (2001). Cannabinoid receptor activation and elevated cyclic AMP reduce glutamate neurotoxicity. Eur. J. Neurosci..

[69] Huang C.C., Chen Y.L., Lo S.W., Hsu K.S. (2002). Activation of cAMP-dependent protein kinase suppresses the presynaptic cannabinoid inhibition of glutamatergic transmission at corticostriatal synapses. Mol. Pharmacol..

[70] Gyombolai P., Boros E., Hunyady L., Turu G. (2013). Differential β-arrestin 2 requirements for constitutive and agonist-induced internalization of the CB1 cannabinoid receptor. Mol. Cell. Endocrinol..

[71] Ahn K.H., Mahmoud M.M., Shim J.-Y., Kendall D.A. (2013). Distinct roles of β-arrestin 1 and β-arrestin 2 in ORG27569-induced biased signaling and internalization of the cannabinoid receptor 1 (CB1). J. Biol. Chem..

[72] Kang K.A., Wang Z.H., Zhang R., Piao M.J., Kim K.C., Kang S.S., Kim Y.W., Lee J., Park D., Hyun J.W. (2010). Myricetin protects cells against oxidative stress-induced apoptosis via regulation of PI3K/Akt and MAPK signaling pathways. Int. J. Mol. Sci..

[73] Jazvinšćak Jembrek M., Radovanović V., Vlainić J., Vuković L., Hanžić N. (2018). Neuroprotective effect of zolpidem against glutamate-induced toxicity is mediated via the PI3K/Akt pathway and inhibited by PK11195. Toxicology.

[74] Jazvinšćak Jembrek M., Vlainić J., Čadež V., Šegota S. (2018). Atomic force microscopy reveals new biophysical markers for monitoring subcellular changes in oxidative injury: Neuroprotective effects of quercetin at the nanoscale. PLoS ONE.

[75] Zubčić K., Radovanović V., Vlainić J., Hof P.R., Oršolić N., Šimić G., Jazvinšćak Jembrek M. (2020). PI3K/Akt and ERK1/2 Signalling Are Involved in Quercetin-Mediated Neuroprotection against Copper-Induced Injury. Oxid. Med. Cell. Longev..

[76] Yang X., Zhang H., Wu J., Yin L., Yan L.J., Zhang C. (2018). Humanin Attenuates NMDA-Induced Excitotoxicity by Inhibiting ROS-dependent JNK/p38 MAPK Pathway. Int. J. Mol. Sci..

[77] Zakharova I.O., Sokolova T.V., Bayunova L.V., Zorina I.I., Rychkova M.P., Shpakov A.O., Avrova N.F. (2019). The Protective Effect of Insulin on Rat Cortical Neurons in Oxidative Stress and Its Dependence on the Modulation of Akt, GSK-3beta, ERK1/2, and AMPK Activities. Int. J. Mol. Sci..

[78] Rueda D., Galve-Roperh I., Haro A., Guzmán M. (2000). The CB(1) cannabinoid receptor is coupled to the activation of c-Jun N-terminal kinase. Mol. Pharmacol..

[79] Davis M.I., Ronesi J., Lovinger D.M. (2003). A predominant role for inhibition of the adenylate cyclase/protein kinase A pathway in ERK activation by cannabinoid receptor 1 in N1E-115 neuroblastoma cells. J. Biol. Chem..

[80] Derkinderen P., Valjent E., Toutant M., Corvol J.C., Enslen H., Ledent C., Trzaskos J., Caboche J., Girault J.A. (2003). Regulation of extracellular signal-regulated kinase by cannabinoids in hippocampus. J. Neuroci..

[81] Bouaboula M., Poinot-Chazel C., Bourrié B., Canat X., Calandra B., Rinaldi-Carmona M., Le Fur G., Casellas P. (1995). Activation of mitogen-activated protein kinases by stimulation of the central cannabinoid receptor CB1. Biochem. J..

[82] Asimaki O., Mangoura D. (2011). Cannabinoid receptor 1 induces a biphasic ERK activation via multiprotein signaling complex formation of proximal kinases PKCε, Src, and Fyn in primary neurons. Neurochem. Int..

[83] Blázquez C., Chiarlone A., Bellocchio L., Resel E., Pruunsild P., García-Rincón D., Sendtner M., Timmusk T., Lutz B., Galve-Roperh I. (2015). The CB1 cannabinoid receptor signals striatal neuroprotection via a PI3K/Akt/mTORC1/BDNF pathway. Cell Death Differ..

[84] Xu Z., Lv X.A., Dai Q., Ge Y.Q., Xu J. (2016). Acute upregulation of neuronal mitochondrial type-1 cannabinoid receptor and it’s role in metabolic defects and neuronal apoptosis after TBI. Mol. Brain.

[85] Viscomi M.T., Oddi S., Latini L., Pasquariello N., Florenzano F., Bernardi G., Molinari M., Maccarrone M. (2009). Selective CB2 receptor agonism protects central neurons from remote axotomy-induced apoptosis through the PI3K/Akt pathway. J. Neurosci..

[86] Gómez Del Pulgar T., De Ceballos M.L., Guzmán M., Velasco G. (2002). Cannabinoids protect astrocytes from ceramide-induced apoptosis through the phosphatidylinositol 3-kinase/protein kinase B pathway. J. Biol. Chem..

[87] Jazvinšćak Jembrek M., Hof P.R., Šimić G. (2015). Ceramides in Alzheimer’s Disease: Key Mediators of Neuronal Apoptosis Induced by Oxidative Stress and Aβ Accumulation. Oxid. Med. Cell. Longev..

[88] Velasco G., Galve-Roperh I., Sánchez C., Blázquez C., Haro A., Guzmán M. (2005). Cannabinoids and ceramide: Two lipids acting hand-by-hand. Life Sci..

[89] Carracedo A., Geelen M.J.H., Diez M., Hanada K., Guzmán M., Velasco G. (2004). Ceramide sensitizes astrocytes to oxidative stress: Protective role of cannabinoids. Biochem. J..

[90] Guo J., Ikeda S.R. (2004). Endocannabinoids modulate N-type calcium channels and G-protein-coupled inwardly rectifying potassium channels via CB1 cannabinoid receptors heterologously expressed in mammalian neurons. Mol. Pharmacol..

[91] Kwan Cheung K.A., Peiris H., Wallace G., Holland O.J., Mitchell M.D. (2019). The Interplay between the Endocannabinoid System, Epilepsy and Cannabinoids. Int. J. Mol. Sci..

[92] Kearn C.S., Blake-Palmer K., Daniel E., Mackie K., Glass M. (2005). Concurrent stimulation of cannabinoid CB1 and dopamine D2 receptors enhances heterodimer formation: A mechanism for receptor cross-talk?. Mol. Pharmacol..

[93] Chiang Y.-C., Lo Y.-N., Chen J.-C. (2013). Crosstalk between dopamine D2 receptors and cannabinoid CB1 receptors regulates CNR1 promoter activity via ERK1/2 signaling. J. Neurochem..

[94] Cinar R., Freund T.F., Katona I., Mackie K., Szucs M. (2008). Reciprocal inhibition of G-protein signaling is induced by CB1 cannabinoid and GABAB receptor interactions in rat hippocampal membranes. Neurochem. Int..

[95] Callén L., Moreno E., Barroso-Chinea P., Moreno-Delgado D., Cortés A., Mallol J., Casadó V., Lanciego J.L., Franco R., Lluis C. (2012). Cannabinoid receptors CB 1 and CB 2 form functional heteromers in brain. J. Biol. Chem..

[96] Bénard G., Massa F., Puente N., Lourenço J., Bellocchio L., Soria-Gómez E., Matias I., Delamarre A., Metna-Laurent M., Cannich A. (2012). Mitochondrial CB₁ receptors regulate neuronal energy metabolism. Nat. Neurosci..

[97] Hebert-Chatelain E., Reguero L., Puente N., Lutz B., Chaouloff F., Rossignol R., Piazza P.V., Benard G., Grandes P., Marsicano G. (2014). Cannabinoid control of brain bioenergetics: Exploring the subcellular localization of the CB1 receptor. Mol. Metab..

[98] Chaturvedi R.K., Beal M.F. (2008). Mitochondrial approaches for neuroprotection. Ann. N. Y. Acad. Sci..

[99] Naoi M., Wu Y., Shamoto-Nagai M., Maruyama W. (2019). Mitochondria in Neuroprotection by Phytochemicals: Bioactive Polyphenols Modulate Mitochondrial Apoptosis System, Function and Structure. Int. J. Mol. Sci..

[100] Ma L., Jia J., Niu W., Jiang T., Zhai Q., Yang L., Bai F., Wang Q., Xiong L. (2015). Mitochondrial CB1 receptor is involved in ACEA-induced protective effects on neurons and mitochondrial functions. Sci. Rep..

[101] Khajehali E., Malone D.T., Glass M., Sexton P.M., Christopoulos A., Leach K. (2015). Biased Agonism and Biased Allosteric Modulation at the CB1 Cannabinoid Receptor. Mol. Pharmacol..

[102] Laprairie R.B., Bagher A.M., Kelly M.E.M., Denovan-Wright E.M. (2016). Biased Type 1 Cannabinoid Receptor Signaling Influences Neuronal Viability in a Cell Culture Model of Huntington Disease. Mol. Pharmacol..

[103] Verma M., Lizama B.N., Chu C.T. (2022). Excitotoxicity, calcium and mitochondria: A triad in synaptic neurodegeneration. Transl. Neurodegener..

[104] Singh A., Kukreti R., Saso L., Kukreti S. (2019). Oxidative Stress: A Key Modulator in Neurodegenerative Diseases. Molecules.

[105] Ramírez B.G., Blázquez C., Gómez del Pulgar T., Guzmán M., de Ceballos M.L. (2005). Prevention of Alzheimer’s disease pathology by cannabinoids: Neuroprotection mediated by blockade of microglial activation. J. Neurosci..

[106] De la Monte S.M., Jhaveri A., Maron B.A., Wands J.R. (2007). Nitric oxide synthase 3-mediated neurodegeneration after intracerebral gene delivery. J. Neuropathol. Exp. Neurol..

[107] Picón-Pagès P., Garcia-Buendia J., Muñoz F.J. (2019). Functions and dysfunctions of nitric oxide in brain. Biochim. Biophys. Acta Mol. Basis Dis..

[108] Ebadi M., Sharma S.K., Ghafourifar P., Brown-Borg H., El Refaey H. (2005). Peroxynitrite in the pathogenesis of Parkinson’s disease and the neuroprotective role of metallothioneins. Methods Enzymol..

[109] Leathem A., Ortiz-Cerda T., Dennis J.M., Witting P.K. (2022). Evidence for Oxidative Pathways in the Pathogenesis of PD: Are Antioxidants Candidate Drugs to Ameliorate Disease Progression?. Int. J. Mol. Sci..

[110] Javed H., Azimullah S., Haque M.E., Ojha S.K. (2016). Cannabinoid Type 2 (CB2) Receptors Activation Protects against Oxidative Stress and Neuroinflammation Associated Dopaminergic Neurodegeneration in Rotenone Model of Parkinson’s Disease. Front. Neurosci..

[111] Bono-Yagüe J., Gómez-Escribano A.P., Millán J.M., Vázquez-Manrique R.P. (2020). Reactive Species in Huntington Disease: Are They Really the Radicals You Want to Catch?. Antioxidants.

[112] Zhao P., Leonoudakis D., Abood M.E., Beattie E.C. (2010). Cannabinoid receptor activation reduces TNFalpha-induced surface localization of AMPAR-type glutamate receptors and excitotoxicity. Neuropharmacology.

[113] Zhuang S.Y., Bridges D., Grigorenko E., McCloud S., Boon A., Hampson R.E., Deadwyler S.A. (2005). Cannabinoids produce neuroprotection by reducing intracellular calcium release from ryanodine-sensitive stores. Neuropharmacology.

[114] Hampson A.J., Bornheim L.M., Scanziani M., Yost C.S., Gray A.T., Hansen B.M., Leonoudakis D.J., Bickler P.E. (1998). Dual effects of anandamide on NMDA receptor-mediated responses and neurotransmission. J. Neurochem..

[115] Khaspekov L.G., Brenz Verca M.S., Frumkina L.E., Hermann H., Marsicano G., Lutz B. (2004). Involvement of brain-derived neurotrophic factor in cannabinoid receptor-dependent protection against excitotoxicity. Eur. J. Neurosci..

[116] Vicente-Sánchez A., Sánchez-Blázquez P., Rodríguez-Muñoz M., Garzón J. (2013). HINT1 protein cooperates with cannabinoid 1 receptor to negatively regulate glutamate NMDA receptor activity. Mol. Brain.

[117] Kreutz S., Koch M., Ghadban C., Korf H.W., Dehghani F. (2007). Cannabinoids and neuronal damage: Differential effects of THC, AEA and 2-AG on activated microglial cells and degenerating neurons in excitotoxically lesioned rat organotypic hippocampal slice cultures. Exp. Neurol..

[118] Kreutz S., Koch M., Böttger C., Ghadban C., Korf H.W., Dehghani F. (2009). 2-Arachidonoylglycerol elicits neuroprotective effects on excitotoxically lesioned dentate gyrus granule cells via abnormal-cannabidiol-sensitive receptors on microglial cells. Glia.

[119] Netzeband J.G., Conroy S.M., Parsons K.L., Gruol D.L. (1999). Cannabinoids enhance NMDA-elicited Ca2+ signals in cerebellar granule neurons in culture. J. Neuroscienci..

[120] Panikashvili D., Shein N.A., Mechoulam R., Trembovler V., Kohen R., Alexandrovich A., Shohami E. (2006). The endocannabinoid 2-AG protects the blood-brain barrier after closed head injury and inhibits mRNA expression of proinflammatory cytokines. Neurobiol. Dis..

[121] Yu S.J., Reiner D., Shen H., Wu K.J., Liu Q.R., Wang Y. (2015). Time-dependent protection of CB2 receptor agonist in stroke. PLoS ONE.

[122] Vasincu A., Rusu R.-N., Ababei D.-C., Larion M., Bild W., Stanciu G.D., Solcan C., Bild V. (2022). Endocannabinoid Modulation in Neurodegenerative Diseases: In Pursuit of Certainty. Biology.

[123] Armeli F., Bonucci A., Maggi E., Pinto A., Businaro R. (2021). Mediterranean Diet and Neurodegenerative Diseases: The Neglected Role of Nutrition in the Modulation of the Endocannabinoid System. Biomolecules.

[124] Ceccarini J., Ahmad R., Van De Vliet L., Casteels C., Vandenbulcke M., Vandenberghe W., Van Laere K. (2019). Behavioral symptoms in premanifest Huntington disease correlate with reduced frontal CB 1 R levels. J. Nucl. Med..

[125] Glass M., Dragunow M., Faull R.L. (2000). The pattern of neurodegeneration in Huntington’s disease: A comparative study of cannabinoid, dopamine, adenosine and GABA(A) receptor alterations in the human basal ganglia in Huntington’s disease. Neuroscience.

[126] Van Laere K., Casteels C., Dhollander I., Goffin K., Grachev I., Bormans G., Vandenberghe W. (2010). Widespread decrease of type 1 cannabinoid receptor availability in Huntington disease in vivo. J. Nucl. Med..

[127] López-Sendón Moreno J.L., García Caldentey J., Trigo Cubillo P., Ruiz Romero C., García Ribas G., Alonso Arias M.A.A., García de Yébenes M.J., Tolón R.M., Galve-Roperh I., Sagredo O. (2016). A double-blind, randomized, cross-over, placebo-controlled, pilot trial with Sativex in Huntington’s disease. J. Neurol..

[128] Lastres-Becker I., Bizat N., Boyer F., Hantraye P., Brouillet E., Fernández-Ruiz J. (2003). Effects of cannabinoids in the rat model of Huntington’s disease generated by an intrastriatal injection of malonate. Neuroreport.

[129] Dhopeshwarkar A., Mackie K. (2016). Functional Selectivity of CB2 Cannabinoid Receptor Ligands at a Canonical and Noncanonical Pathway. J. Pharm. Exp. Ther..

[130] Bedse G., Romano A., Cianci S., Lavecchia A.M., Lorenzo P., Elphick M.R., Laferla F.M., Vendemiale G., Grillo C., Altieri F. (2014). Altered expression of the CB1 cannabinoid receptor in the triple transgenic mouse model of Alzheimer’s disease. J. Alzheimers Dis..

[131] Mulder J., Zilberter M., Pasquaré S.J., Alpár A., Schulte G., Ferreira S.G., Köfalvi A., Martín-Moreno A.M., Keimpema E., Tanila H. (2011). Molecular reorganization of endocannabinoid signalling in Alzheimer’s disease. Brain.

[132] Pascual A.C., Martín-Moreno A.M., Giusto N.M., de Ceballos M.L., Pasquaré S.J. (2014). Normal aging in rats and pathological aging in human Alzheimer’s disease decrease FAAH activity: Modulation by cannabinoid agonists. Exp. Gerontol..

[133] Jung K.M., Astarita G., Yasar S., Vasilevko V., Cribbs D.H., Head E., Cotman C.W., Piomelli D. (2012). An amyloid β42-dependent deficit in anandamide mobilization is associated with cognitive dysfunction in Alzheimer’s disease. Neurobiol. Aging.

[134] Solas M., Francis P.T., Franco R., Ramirez M.J. (2013). CB2 receptor and amyloid pathology in frontal cortex of Alzheimer’s disease patients. Neurobiol. Aging.

[135] Wu J., Bie B., Yang H., Xu J.J., Brown D.L., Naguib M. (2013). Activation of the CB2 receptor system reverses amyloid-induced memory deficiency. Neurobiol. Aging.

[136] Stumm C., Hiebel C., Hanstein R., Purrio M., Nagel H., Conrad A., Lutz B., Behl C., Clement A.B. (2013). Cannabinoid receptor 1 deficiency in a mouse model of Alzheimer’s disease leads to enhanced cognitive impairment despite of a reduction in amyloid deposition. Neurobiol. Aging.

[137] Altamura C., Ventriglia M., Martini M.G., Montesano D., Errante Y., Piscitelli F., Scrascia F., Quattrocchi C., Palazzo P., Seccia S. (2015). Elevation of Plasma 2-Arachidonoylglycerol Levels in Alzheimer’s Disease Patients as a Potential Protective Mechanism against Neurodegenerative Decline. J. Alzheimers Dis..

[138] Van der Stelt M., Mazzola C., Esposito G., Matias I., Petrosino S., De Filippis D., Micale V., Steardo L., Drago F., Iuvone T. (2006). Endocannabinoids and beta-amyloid-induced neurotoxicity in vivo: Effect of pharmacological elevation of endocannabinoid levels. Cell. Mol. Life Sci..

[139] Chen R., Zhang J., Wu Y., Wang D., Feng G., Tang Y.P., Teng Z., Chen C. (2012). Monoacylglycerol lipase is a therapeutic target for Alzheimer’s disease. Cell Rep..

[140] Pihlaja R., Takkinen J., Eskola O., Vasara J., López-Picón F.R., Haaparanta-Solin M., Rinne J.O. (2015). Monoacylglycerol lipase inhibitor JZL184 reduces neuroinflammatory response in APdE9 mice and in adult mouse glial cells. J. Neuroinflammation.

[141] Elmazoglu Z., Rangel-López E., Medina-Campos O.N., Pedraza-Chaverri J., Túnez I., Aschner M., Santamaría A., Karasu Ç. (2020). Cannabinoid-profiled agents improve cell survival via reduction of oxidative stress and inflammation, and Nrf2 activation in a toxic model combining hyperglycemia+Aβ_1-42_ peptide in rat hippocampal neurons. Neurochem. Int..

[142] Lastres-Becker I., Cebeira M., De Ceballos M.L., Zeng B.Y., Jenner P., Ramos J.A., Fernández-Ruiz J.J. (2001). Increased cannabinoid CB1 receptor binding and activation of GTP-binding proteins in the basal ganglia of patients with Parkinson’s syndrome and of MPTP-treated marmosets. Eur. J. Neurosci..

[143] Pisani V., Moschella V., Bari M., Fezza F., Galati S., Bernardi G., Stanzione P., Pisani A., Maccarrone M. (2010). Dynamic changes of anandamide in the cerebrospinal fluid of Parkinson’s disease patients. Mov. Disord..

[144] Lotan I., Treves T.A., Roditi Y., Djaldetti R. (2014). Cannabis (medical marijuana) treatment for motor and non-motor symptoms of Parkinson disease: An open-label observational study. Clin. Neuropharmacol..

[145] Chung Y.C., Shin W.H., Baek J.Y., Cho E.J., Baik H.H., Kim S.R., Won S.Y., Jin B.K. (2016). CB2 receptor activation prevents glial-derived neurotoxic mediator production, BBB leakage and peripheral immune cell infiltration and rescues dopamine neurons in the MPTP model of Parkinson’s disease. Exp. Mol. Med..

[146] Witting A., Weydt P., Hong S., Kliot M., Moller T., Stella N. (2004). Endocannabinoids accumulate in spinal cord of SOD1 G93A transgenic mice. J. Neurochem..

[147] Yiangou Y., Facer P., Durrenberger P., Chessell I.P., Naylor A., Bountra C., Banati R.R., Anand P. (2006). COX-2, CB2 and P2X7-immunoreactivities are increased in activated microglial cells/macrophages of multiple sclerosis and amyotrophic lateral sclerosis spinal cord. BMC Neurol..

[148] Romigi A., Bari M., Placidi F., Marciani M.G., Malaponti M., Torelli F., Izzi F., Prosperetti C., Zannino S., Corte F. (2010). Cerebrospinal fluid levels of the endocannabinoid anandamide are reduced in patients with untreated newly diagnosed temporal lobe epilepsy. Epilepsia.

[149] Rocha L., Cinar R., Guevara-Guzmán R., Alonso-Vanegas M., San-Juan D., Martínez-Juárez I., Castañeda-Cabral J.L., Carmona-Cruz F. (2020). Endocannabinoid System and Cannabinoid 1 Receptors in Patients With Pharmacoresistant Temporal Lobe Epilepsy and Comorbid Mood Disorders. Front. Behav. Neurosci..

[150] Ludányi A., Eross L., Czirják S., Vajda J., Halász P., Watanabe M., Palkovits M., Maglóczky Z., Freund T.F., Katona I. (2008). Downregulation of the CB1 cannabinoid receptor and related molecular elements of the endocannabinoid system in epileptic human hippocampus. J. Neurosci..

[151] Perucca E. (2017). Cannabinoids in the Treatment of Epilepsy: Hard Evidence at Last?. J. Epilepsy Res..

[152] Funada M., Takebayashi-Ohsawa M. (2018). Synthetic cannabinoid AM2201 induces seizures: Involvement of cannabinoid CB_1_ receptors and glutamatergic transmission. Toxicol. Appl. Pharmacol..

[153] Knowles M.D., de la Tremblaye P.B., Azogu I., Plamondon H. (2016). Endocannabinoid CB1 receptor activation upon global ischemia adversely impact recovery of reward and stress signaling molecules, neuronal survival and behavioral impulsivity. Prog. Neuro-Psychopharmacol. Biol. Psychiatry.

[154] Lopez-Rodriguez A.B., Siopi E., Finn D.P., Marchand-Leroux C., Garcia-Segura L.M., Jafarian-Tehrani M., Viveros M.P. (2015). CB1 and CB2 cannabinoid receptor antagonists prevent minocycline-induced neuroprotection following traumatic brain injury in mice. Cereb. Cortex..

[155] Behl T., Makkar R., Sehgal A., Singh S., Sharma N., Zengin G., Bungau S., Andronie-Cioara F.L., Munteanu M.A., Brisc M.C. (2021). Current trends in neurodegeneration: Cross talks between oxidative stress, cell death, and inflammation. Int. J. Mol. Sci..

[156] Liu Z., Zhou T., Ziegler A.C., Dimitrion P., Zuo L. (2017). Oxidative stress in neurodegenerative diseases: From molecular mechanisms to clinical applications. Oxid. Med. Cell. Long..

[157] Jazvinšćak Jembrek M., Oršolić N., Mandić L., Sadžak A., Šegota S. (2021). Anti-Oxidative, Anti-Inflammatory and Anti-Apoptotic Effects of Flavonols: Targeting Nrf2, NF-κB and p53 Pathways in Neurodegeneration. Antioxidants.

[158] Domanskyi A., Parlato R. (2022). Oxidative Stress in Neurodegenerative Diseases. Antioxidants.

[159] Piccirillo S., Magi S., Preziuso A., Serfilippi T., Cerqueni G., Orciani M., Amoroso S., Lariccia V. (2022). The Hidden Notes of Redox Balance in Neurodegenerative Diseases. Antioxidants.

[160] Ashok A., Andrabi S.S., Mansoor S., Kuang Y., Kwon B.K., Labhasetwar V. (2022). Antioxidant Therapy in Oxidative Stress-Induced Neurodegenerative Diseases: Role of Nanoparticle-Based Drug Delivery Systems in Clinical Translation. Antioxidants.

[161] Cobley J.N., Fiorello M.L., Bailey D.M. (2018). 13 reasons why the brain is susceptible to oxidative stress. Redox Biol..

[162] Zubčić K., Hof P.R., Šimić G., Jazvinšćak Jembrek M. (2020). The Role of Copper in Tau-Related Pathology in Alzheimer’s Disease. Front. Mol. Neurosci..

[163] Shoeb M., Ansari N.H., Srivastava S.K., Ramana K.V. (2014). 4-Hydroxynonenal in the pathogenesis and progression of human diseases. Curr. Med. Chem..

[164] Petrovic S., Arsic A., Ristic-Medic D., Cvetkovic Z., Vucic V. (2020). Lipid Peroxidation and Antioxidant Supplementation in Neurodegenerative Diseases: A Review of Human Studies. Antioxidants.

[165] Teleanu D.M., Niculescu A.-G., Lungu I.I., Radu C.I., Vladâcenco O., Roza E., Costăchescu B., Grumezescu A.M., Teleanu R.I. (2022). An Overview of Oxidative Stress, Neuroinflammation, and Neurodegenerative Diseases. Int. J. Mol. Sci..

[166] Rauf A., Badoni H., Abu-Izneid T., Olatunde A., Rahman M.M., Painuli S., Semwal P., Wilairatana P., Mubarak M.S. (2022). Neuroinflammatory Markers: Key Indicators in the Pathology of Neurodegenerative Diseases. Molecules.

[167] De Oliveira J., Kucharska E., Garcez M.L., Rodrigues M.S., Quevedo J., Moreno-Gonzalez I., Budni J. (2021). Inflammatory Cascade in Alzheimer’s Disease Pathogenesis: A Review of Experimental Findings. Cells.

[168] Kosyreva A.M., Sentyabreva A.V., Tsvetkov I.S., Makarova O.V. (2022). Alzheimer’s Disease and Inflammaging. Brain Sci..

[169] Picca A., Calvani R., Coelho-Junior H.J., Landi F., Bernabei R., Marzetti E. (2020). Mitochondrial Dysfunction, Oxidative Stress, and Neuroinflammation: Intertwined Roads to Neurodegeneration. Antioxidants.

[170] Buccellato F.R., D’Anca M., Fenoglio C., Scarpini E., Galimberti D. (2021). Role of Oxidative Damage in Alzheimer’s Disease and Neurodegeneration: From Pathogenic Mechanisms to Biomarker Discovery. Antioxidants.

[171] Ryan K.C., Ashkavand Z., Norman K.R. (2020). The Role of Mitochondrial Calcium Homeostasis in Alzheimer’s and Related Diseases. Int. J. Mol. Sci..

[172] Eshraghi M., Adlimoghaddam A., Mahmoodzadeh A., Sharifzad F., Yasavoli-Sharahi H., Lorzadeh S., Albensi B.C., Ghavami S. (2021). Alzheimer’s Disease Pathogenesis: Role of Autophagy and Mitophagy Focusing in Microglia. Int. J. Mol. Sci..

[173] Woo J., Cho H., Seol Y., Kim S.H., Park C., Yousefian-Jazi A., Hyeon S.J., Lee J., Ryu H. (2021). Power Failure of Mitochondria and Oxidative Stress in Neurodegeneration and Its Computational Models. Antioxidants.

[174] Musetti B., González-Ramos H., González M., Bahnson E.M., Varela J., Thomson L. (2020). Cannabis sativa extracts protect LDL from Cu^2+^-mediated oxidation. J. Cannabis Res..

[175] Borges R.S., Batista J., Viana R.B., Baetas A.C., Orestes E., Andrade M.A., Honório K.M., Da Silva A.B.F. (2013). Understanding the Molecular Aspects of Tetrahydrocannabinol and Cannabidiol as Antioxidants. Molecules.

[176] Duncan R.S., Riordan S.M., Hall C.W., Payne A.J., Chapman K.D., Koulen P. (2022). *N*-acylethanolamide metabolizing enzymes are upregulated in human neural progenitor-derived neurons exposed to sub-lethal oxidative stress. Front. Cell Neurosci..

[177] Parga J.A., Rodriguez-Perez A.I., Garcia-Garrote M., Rodriguez-Pallares J., Labandeira-Garcia J.L. (2021). NRF2 Activation and Downstream Effects: Focus on Parkinson’s Disease and Brain Angiotensin. Antioxidants.

[178] Sivandzade F., Prasad S., Bhalerao A., Cucullo L. (2019). NRF2 and NF-ҡB interplay in cerebrovascular and neurodegenerative disorders: Molecular mechanisms and possible therapeutic approaches. Redox Biol..

[179] Villavicencio Tejo F., Quintanilla R.A. (2021). Contribution of the Nrf2 Pathway on Oxidative Damage and Mitochondrial Failure in Parkinson and Alzheimer’s Disease. Antioxidants.

[180] Galán-Ganga M., Del Río R., Jiménez-Moreno N., Díaz-Guerra M., Lastres-Becker I. (2020). Cannabinoid CB_2_ Receptor Modulation by the Transcription Factor NRF2 is Specific in Microglial Cells. Cell. Mol. Neurobiol..

[181] Chianese G., Sirignano C., Benetti E., Marzaroli V., Collado J.A., de la Vega L., Appendino G., Muñoz E., Taglialatela-Scafati O. (2022). A Nrf-2 Stimulatory Hydroxylated Cannabidiol Derivative from Hemp (*Cannabis sativa*). J. Nat. Prod..

[182] Hampson A.J., Grimaldi M., Axelrod J., Wink D. (1998). Cannabidiol and (-)Delta9-tetrahydrocannabinol are neuroprotective antioxidants. Proc. Natl. Acad. Sci. USA.

[183] Jia J., Ma L., Wu M., Zhang L., Zhang X., Zhai Q., Jiang T., Wang Q., Xiong L. (2014). Anandamide protects HT22 cells exposed to hydrogen peroxide by inhibiting CB1 receptor-mediated type 2 NADPH oxidase. Oxid. Med. Cell. Longev..

[184] Shouman B., Fontaine R.H., Baud O., Schwendimann L., Keller M., Spedding M., Lelièvre V., Gressens P. (2006). Endocannabinoids potently protect the newborn brain against AMPA-kainate receptor-mediated excitotoxic damage. Br. J. Pharmacol..

[185] Liu X., Wu Y., Zhou D., Xie Y., Zhou Y., Lu Y., Yang R., Liu S. (2020). N-linoleyltyrosine protects PC12 cells against oxidative damage via autophagy: Possible involvement of CB1 receptor regulation. Int. J. Mol. Med..

[186] Zhou Y., Li Z.X., Liu Y.T., Xu Z.C., Hu Y., Lv W., Yang Z.Y., Sheng Y.M., Liu S. (2022). N-linoleyltyrosine protects neurons against Aβ_1-40_-induced cell toxicity via autophagy involving the CB_2_/AMPK/mTOR/ULK1 pathway. Brain Res. Bull..

[187] Veeraraghavan P., Dekanic A., Nistri A. (2016). A study of cannabinoid-1 receptors during the early phase of excitotoxic damage to rat spinal locomotor networks in vitro. Neuroscience.

[188] Hassan F.-u., Nadeem A., Li Z., Javed M., Liu Q., Azhar J., Rehman M.S.-u., Cui K., Rehman S.u. (2021). Role of Peroxisome Proliferator-Activated Receptors (PPARs) in Energy Homeostasis of Dairy Animals: Exploiting Their Modulation through Nutrigenomic Interventions. Int. J. Mol. Sci..

[189] Grabacka M., Pierzchalska M., Płonka P.M., Pierzchalski P. (2021). The Role of PPAR Alpha in the Modulation of Innate Immunity. Int. J. Mol. Sci..

[190] Zhou Y., Yang L., Ma A., Zhang X., Li W., Yang W., Chen C., Jin X. (2012). Orally administered oleoylethanolamide protects mice from focal cerebral ischemic injury by activating peroxisome proliferator-activated receptor α. Neuropharmacology.

[191] Panikashvili D., Simeonidou C., Ben-Shabat S., Hanus L., Breuer A., Mechoulam R., Shohami E. (2001). An endogenous cannabinoid (2-AG) is neuroprotective after brain injury. Nature.

[192] Panikashvili D., Mechoulam R., Beni S.M., Alexandrovich A., Shohami E. (2005). CB1 cannabinoid receptors are involved in neuroprotection via NF-kappa B inhibition. J. Cereb. Blood Flow Metab..

[193] Zhang J., Teng Z., Song Y., Hu M., Chen C. (2015). Inhibition of monoacylglycerol lipase prevents chronic traumatic encephalopathy-like neuropathology in a mouse model of repetitive mild closed head injury. J. Cereb. Blood Flow Metab..

[194] Aymerich M.S., Rojo-Bustamante E., Molina C., Celorrio M., Sánchez-Arias J.A., Franco R. (2016). Neuroprotective Effect of JZL184 in MPP(+)-Treated SH-SY5Y Cells Through CB2 Receptors. Mol. Neurobiol..

[195] Tchantchou F., Tucker L.B., Fu A.H., Bluett R.J., McCabe J.T., Patel S., Zhang Y. (2014). The fatty acid amide hydrolase inhibitor PF-3845 promotes neuronal survival, attenuates inflammation and improves functional recovery in mice with traumatic brain injury. Neuropharmacology.

[196] Bilsland L.G., Dick J.R., Pryce G., Petrosino S., Di Marzo V., Baker D., Greensmith L. (2006). Increasing cannabinoid levels by pharmacological and genetic manipulation delay disease progression in SOD1 mice. FASEB J..

[197] Celorrio M., Fernández-Suárez D., Rojo-Bustamante E., Echeverry-Alzate V., Ramírez M.J., Hillard C.J., López-Moreno J.A., Maldonado R., Oyarzábal J., Franco R. (2016). Fatty acid amide hydrolase inhibition for the symptomatic relief of Parkinson’s disease. Brain Behav. Immun..

[198] Bergamaschi M.M., Queiroz R.H., Zuardi A.W., Crippa J.A. (2011). Safety and side effects of cannabidiol, a Cannabis sativa constituent. Curr. Drug Saf..

[199] Iffland K., Grotenhermen F. (2016). An Update on Safety and Side Effects of Cannabidiol: A Review of Clinical Data and Relevant Animal Studies. Cannabis Cannabinoid Res..

[200] Bisogno T., Hanus L., De Petrocellis L., Tchilibon S., Ponde D.E., Brandi I., Moriello A.S., Davis J.B., Mechoulam R., Di Marzo V. (2001). Molecular targets for cannabidiol and its synthetic analogues: Effect on vanilloid VR1 receptors and on the cellular uptake and enzymatic hydrolysis of anandamide. Br. J. Pharmacol..

[201] Papagianni E.P., Stevenson C.W. (2019). Cannabinoid Regulation of Fear and Anxiety: An Update. Curr. Psychiatry Rep..

[202] García-Gutiérrez M.S., Navarrete F., Gasparyan A., Austrich-Olivares A., Sala F., Manzanares J. (2020). Cannabidiol: A Potential New Alternative for the Treatment of Anxiety, Depression, and Psychotic Disorders. Biomolecules.

[203] Campos A.C., Fogaça M.V., Sonego A.B., Guimarães F.S. (2016). Cannabidiol, neuroprotection and neuropsychiatric disorders. Pharmacol. Res..

[204] Gunasekera B., Davies C., Blest-Hopley G., Veronese M., Ramsey N.F., Bossong M.G., Radua J., Bhattacharyya S., CBE Consortium (2022). Task-independent acute effects of delta-9-tetrahydrocannabinol on human brain function and its relationship with cannabinoid receptor gene expression: A neuroimaging meta-regression analysis. Neurosci. Biobehav. Rev..

[205] Kopustinskiene D.M., Masteikova R., Lazauskas R., Bernatoniene J. (2022). *Cannabis sativa* L. Bioactive Compounds and Their Protective Role in Oxidative Stress and Inflammation. Antioxidants.

[206] Nadal X., Del Río C., Casano S., Palomares B., Ferreiro-Vera C., Navarrete C., Sánchez-Carnerero C., Cantarero I., Bellido M.L., Meyer S. (2017). Tetrahydrocannabinolic acid is a potent PPARγ agonist with neuroprotective activity. Br. J. Pharmacol..

[207] Verhoeckx K.C., Korthout H.A., van Meeteren-Kreikamp A.P., Ehlert K.A., Wang M., van der Greef J., Rodenburg R.J., Witkamp R.F. (2006). Unheated Cannabis sativa extracts and its major compound THC-acid have potential immuno-modulating properties not mediated by CB1 and CB2 receptor coupled pathways. Int. Immunopharmacol..

[208] Mecha M., Torrao A.S., Mestre L., Carrillo-Salinas F.J., Mechoulam R., Guaza C. (2012). Cannabidiol protects oligodendrocyte progenitor cells from inflammation-induced apoptosis by attenuating endoplasmic reticulum stress. Cell Death Dis..

[209] Castillo A., Tolón M.R., Fernández-Ruiz J., Romero J., Martinez-Orgado J. (2010). The neuroprotective effect of cannabidiol in an in vitro model of newborn hypoxic-ischemic brain damage in mice is mediated by CB(2) and adenosine receptors. Neurobiol. Dis..

[210] Vrechi T., Leão A., Morais I., Abílio V.C., Zuardi A.W., Hallak J., Crippa J.A., Bincoletto C., Ureshino R.P., Smaili S.S. (2021). Cannabidiol induces autophagy via ERK1/2 activation in neural cells. Sci. Rep..

[211] Iuvone T., Esposito G., Esposito R., Santamaria R., Di Rosa M., Izzo A.A. (2004). Neuroprotective effect of cannabidiol, a non-psychoactive component from Cannabis sativa, on beta-amyloid-induced toxicity in PC12 cells. J. Neurochem..

[212] Esposito G., De Filippis D., Carnuccio R., Izzo A.A., Iuvone T. (2006). The marijuana component cannabidiol inhibits beta-amyloid-induced tau protein hyperphosphorylation through Wnt/beta-catenin pathway rescue in PC12 cells. J. Mol. Med..

[213] Esposito G., De Filippis D., Maiuri M.C., De Stefano D., Carnuccio R., Iuvone T. (2006). Cannabidiol inhibits inducible nitric oxide synthase protein expression and nitric oxide production in beta-amyloid stimulated PC12 neurons through p38 MAP kinase and NF-kappaB involvement. Neurosci. Lett..

[214] Esposito G., Scuderi C., Valenza M., Togna G.I., Latina V., De Filippis D., Cipriano M., Carratù M.R., Iuvone T., Steardo L. (2011). Cannabidiol reduces Aβ-induced neuroinflammation and promotes hippocampal neurogenesis through PPARγ involvement. PLoS ONE.

[215] Esposito G., Scuderi C., Savani C., Steardo L., De Filippis D., Cottone P., Iuvone T., Cuomo V., Steardo L. (2007). Cannabidiol in vivo blunts beta-amyloid induced neuroinflammation by suppressing IL-1beta and iNOS expression. Br. J. Pharmacol..

[216] Martín-Moreno A.M., Reigada D., Ramírez B.G., Mechoulam R., Innamorato N., Cuadrado A., de Ceballos M.L. (2011). Cannabidiol and other cannabinoids reduce microglial activation in vitro and in vivo: Relevance to Alzheimer’s disease. Mol. Pharmacol..

[217] Cao C., Li Y., Liu H., Bai G., Mayl J., Lin X., Sutherland K., Nabar N., Cai J. (2014). The potential therapeutic effects of THC on Alzheimer’s disease. J. Alzheimers Dis..

[218] Wang Y., Hong Y., Yan J., Brown B., Lin X., Zhang X., Shen N., Li M., Cai J., Gordon M. (2022). Low-Dose Delta-9-Tetrahydrocannabinol as Beneficial Treatment for Aged APP/PS1 Mice. Int. J. Mol. Sci..

[219] Fihurka O., Hong Y., Yan J., Brown B., Lin X., Shen N., Wang Y., Zhao H., Gordon M.N., Morgan D. (2022). The Memory Benefit to Aged APP/PS1 Mice from Long-Term Intranasal Treatment of Low-Dose THC. Int. J. Mol. Sci..

[220] Lastres-Becker I., Molina-Holgado F., Ramos J.A., Mechoulam R., Fernández-Ruiz J. (2005). Cannabinoids provide neuroprotection against 6-hydroxydopamine toxicity in vivo and in vitro: Relevance to Parkinson’s disease. Neurobiol. Dis..

[221] García-Arencibia M., González S., de Lago E., Ramos J.A., Mechoulam R., Fernández-Ruiz J. (2007). Evaluation of the neuroprotective effect of cannabinoids in a rat model of Parkinson’s disease: Importance of antioxidant and cannabinoid receptor-independent properties. Brain Res..

[222] García C., Palomo-Garo C., García-Arencibia M., Ramos J., Pertwee R., Fernández-Ruiz J. (2011). Symptom-relieving and neuroprotective effects of the phytocannabinoid Δ⁹-THCV in animal models of Parkinson’s disease. Br. J. Pharmacol..

[223] Sagredo O., Ramos J.A., Decio A., Mechoulam R., Fernández-Ruiz J. (2007). Cannabidiol reduced the striatal atrophy caused 3-nitropropionic acid in vivo by mechanisms independent of the activation of cannabinoid, vanilloid TRPV1 and adenosine A2A receptors. Eur. J. Neurosci..

[224] Erukainure O.L., Matsabisa M.G., Salau V.F., Islam M.S. (2020). Tetrahydrocannabinol-Rich Extracts from *Cannabis sativa* L. Improve Glucose Consumption and Modulate Metabolic Complications Linked to Neurodegenerative Diseases in Isolated Rat Brains. Front. Pharmacol..

[225] Valdeolivas S., Satta V., Pertwee R.G., Fernández-Ruiz J., Sagredo O. (2012). Sativex-like combination of phytocannabinoids is neuroprotective in malonate-lesioned rats, an inflammatory model of Huntington’s disease: Role of CB1 and CB2 receptors. ACS Chem. Neurosci..

[226] Moreno-Martet M., Espejo-Porras F., Fernández-Ruiz J., de Lago E. (2014). Changes in endocannabinoid receptors and enzymes in the spinal cord of SOD1(G93A) transgenic mice and evaluation of a Sativex(®) -like combination of phytocannabinoids: Interest for future therapies in amyotrophic lateral sclerosis. CNS Neurosci. Ther..

[227] Amtmann D., Weydt P., Johnson K.L., Jensen M.P., Carter G.T. (2004). Survey of cannabis use in patients with amyotrophic lateral sclerosis. Am. J. Hosp. Paliat. Care.

[228] Paes-Colli Y., Aguiar A., Isaac A.R., Ferreira B.K., Campos R., Trindade P., de Melo Reis R.A., Sampaio L.S. (2022). Phytocannabinoids and Cannabis-Based Products as Alternative Pharmacotherapy in Neurodegenerative Diseases: From Hypothesis to Clinical Practice. Front. Cell. Neurosci..

[229] Kim S.H., Won S.J., Mao X.O., Jin K., Greenberg D.A. (2005). Involvement of protein kinase A in cannabinoid receptor-mediated protection from oxidative neuronal injury. J. Pharmacol. Exp. Ther..

[230] Kim S.H., Won S.J., Mao X.O., Jin K., Greenberg D.A. (2006). Molecular mechanisms of cannabinoid protection from neuronal excitotoxicity. Mol. Pharmacol..

[231] Colín-González A.L., Paz-Loyola A.L., Serratos I.N., Seminotti B., Ribeiro C.A., Leipnitz G., Souza D.O., Wajner M., Santamaría A. (2015). The effect of WIN 55,212-2 suggests a cannabinoid-sensitive component in the early toxicity induced by organic acids accumulating in glutaric acidemia type I and in related disorders of propionate metabolism in rat brain synaptosomes. Neuroscience.

[232] Liu Q., Bhat M., Bowen W.D., Cheng J. (2009). Signaling pathways from cannabinoid receptor-1 activation to inhibition of N-methyl-D-aspartic acid mediated calcium influx and neurotoxicity in dorsal root ganglion neurons. J. Pharmacol. Exp. Ther..

[233] Rycroft B.K., Gibb A.J. (2004). Inhibitory interactions of calcineurin (phosphatase 2B) and calmodulin on rat hippocampal NMDA receptors. Neuropharmacology.

[234] Martín-Moreno A.M., Brera B., Spuch C., Carro E., García-García L., Delgado M., Pozo M.A., Innamorato N.G., Cuadrado A., de Ceballos M.L. (2012). Prolonged oral cannabinoid administration prevents neuroinflammation, lowers β-amyloid levels and improves cognitive performance in Tg APP 2576 mice. J. Neuroinflammation.

[235] Song L., Yang X., Ma Y., Wu N., Liu Z. (2014). The CB1 cannabinoid receptor agonist reduces L-DOPA-induced motor fluctuation and ERK1/2 phosphorylation in 6-OHDA-lesioned rats. Drug Des. Devel. Ther..

[236] Chung Y.C., Bok E., Huh S.H., Park J.Y., Yoon S.H., Kim S.R., Kim Y.S., Maeng S., Park S.H., Jin B.K. (2011). Cannabinoid receptor type 1 protects nigrostriatal dopaminergic neurons against MPTP neurotoxicity by inhibiting microglial activation. J. Immunol..

[237] Price D.A., Martinez A.A., Seillier A., Koek W., Acosta Y., Fernandez E., Strong R., Lutz B., Marsicano G., Roberts J.L. (2009). WIN55,212-2, a cannabinoid receptor agonist, protects against nigrostriatal cell loss in the 1-methyl-4-phenyl-1,2,3,6-tetrahydropyridine mouse model of Parkinson’s disease. Eur. J. Neurosci..

[238] Shoemaker J.L., Seely K.A., Reed R.L., Crow J.P., Prather P.L. (2007). The CB2 cannabinoid agonist AM-1241 prolongs survival in a transgenic mouse model of amyotrophic lateral sclerosis when initiated at symptom onset. J. Neurochem..

[239] Zhao J., Wang M., Liu W., Ma Z., Wu J. (2020). Activation of cannabinoid receptor 2 protects rat hippocampal neurons against Aβ-induced neuronal toxicity. Neurosci. Lett..

[240] Eshhar N., Striem S., Kohen R., Tirosh O., Biegon A. (1995). Neuroprotective and antioxidant activities of HU-211, a novel NMDA receptor antagonist. Eur. J. Pharmacol..

[241] Nuñez-Borque E., González-Naranjo P., Bartolomé F., Alquézar C., Reinares-Sebastián A., Pérez C., Ceballos M.L., Páez J.A., Campillo N.E., Martín-Requero Á. (2020). Targeting Cannabinoid Receptor Activation and BACE-1 Activity Counteracts TgAPP Mice Memory Impairment and Alzheimer’s Disease Lymphoblast Alterations. Mol. Neurobiol..

[242] Li L., Xu Y., Zhao M., Gao Z. (2020). Neuro-protective roles of long non-coding RNA MALAT1 in Alzheimer’s disease with the involvement of the microRNA-30b/CNR1 network and the following PI3K/AKT activation. Exp. Mol. Pathol..

